# Prognostic and Predictive Value of SARIFA-status Within Molecular Subgroups of Colorectal Cancer: Insights From the Netherlands Cohort Study

**DOI:** 10.1097/PAS.0000000000002408

**Published:** 2025-05-09

**Authors:** Nic G. Reitsam, Kelly Offermans, Colinda C.J.M. Simons, Bianca Grosser, Jessica Zimmermann, Heike I. Grabsch, Bruno Märkl, Piet A. van den Brandt

**Affiliations:** * Pathology, Medical Faculty, University of Augsburg; † Bavarian Cancer Research Center (BZKF), Augsburg, Germany; ‡ Department of Epidemiology, GROW Research Institute for Oncology and Reproduction; § Department of Pathology, GROW Research Institute for Oncology and Reproduction, Maastricht University Medical Center+, Maastricht, the Netherlands; ‖ Pathology and Data Analytics, Leeds Institute of Medical Research at St James’s, University of Leeds, Leeds, UK; ¶ Department of Epidemiology, Care and Public Health Research Institute (CAPHRI), Maastricht University, the Netherlands﻿

**Keywords:** colorectal cancer, SARIFA, tumor-adipocyte interaction

## Abstract

We recently proposed Stroma AReactive Invasion Front Areas (SARIFA), defined as direct tumor-adipocyte interaction at the invasion front, as a novel hematoxylin-and-eosin (H&E)-based histopathological﻿ prognostic biomarker in various cancers. Given that microsatellite instability, *BRAF*, and *RAS* mutation status are routinely tested﻿ for colorectal cancers (CRC), studying SARIFA’s additional prognostic value within these molecular subgroups is crucial. In addition, exploring whether the ﻿survival benefit from adjuvant therapy differs according to SARIFA-status may enhance patient treatment and outcome. SARIFA-status, *BRAF*, *RAS*, and DNA mismatch repair (﻿MMR) status were available for 1726 CRC patients from the prospective Netherlands Cohort Study (NLCS, 1986–2006). In this study, we investigated (1) the relationship between SARIFA-status and CRC﻿ molecular characteristics, (2) the prognostic value of SARIFA-status within these molecular subgroups, and (3) whether SARIFA-status wa﻿s associated with survival benefit from adjuvant therapy. SARIFA-positive CRCs more frequently showed a *BRAF* mutation compared to SARIFA-negative CRCs (*P*<0.001). *BRAF*-mutant/MMR-proficient CRCs were enriched in SARIFA-positive cases. SARIFA-positivity was associated with poor CRC-specific (HR_range_: 1.47 to 1.78) and overall survival (HR_range_: 1.35 to 1.70) within all molecular subgroups except MMR-deficient CRCs. Patients with SARIFA-positive CRC showed a ﻿CRC-specific survival benefit from adjuvant therapy compared to surgery alone (HR_CRC-specific_: 0.59; 95% CI: 0.44-0.79), while no CRC-specific survival benefit was observed for patients with SARIFA-negative CRC. To conclude, our results indicate that SARIFA-positivity is more common in the aggressive subset of *BRAF*-mutant and *BRAF*-mutant/MMR-proficient CRCs. Moreover﻿, SARIFA-positivity provides additional prognostic value within molecular subgroups based on *BRAF*, *RAS*, and MMR status, suggesting that it may enhance prognostic stratification of CRC patients.

With more than 1 million new cases every year, colorectal cancer (CRC) is the third most common cancer globally, and the second leading cause of cancer death.^1^ In particular, the incidence of early-onset CRC in younger patients is rising.^2^ Large clinical trials and novel molecular techniques have significantly improved our understanding of CRC as clinically as well as biologically heterogeneous disease with different molecular subtypes.^3,4^ Based on these new understandings, tailored treatment approaches, such as immunotherapy in microsatellite-instable/deficient ﻿mismatch repair (MSI/dMMR) CRC ﻿or anti-EGFR inhibition in *RAS* wild-type (*KRAS* and *NRAS* exon 2-4) CRC, are nowadays applied in the clinic.^5^ Hence, routine MSI, *BRAF*, and *RAS (KRAS/NRAS*) testing has been implemented in diagnostic practice for locally advanced or metastatic CRC in many health care systems.^5,6^

Nevertheless, disease staging according to AJCC/UICC/tumor-node-metastasis (TNM) remains a cornerstone for guiding therapeutic decisions in CRC patients, especially in the adjuvant setting. However, beyond pTNM staging, which has proven prognostic value, the evaluation of other histologic parameters might improve CRC patient prognostication and difficult patient management decisions.^7^ Even though novel RNA-based subtyping approaches, such as consensus molecular subtypes (CMS) or pathway-derived subtyping (PDS),^8,9^ have shown promising results,^10–12^ none of these novel approaches has so far been implemented into routine diagnostics as they are not easily applicable as well as time- and cost-intensive.

We recently proposed Stroma AReactive Invasion Front Areas (SARIFA) as a novel easy-to-implement hematoxylin-and-eosin (H&E)-based histopathological prognostic biomarker in various cancer entities.^13–18^ SARIFA, defined as direct tumor-adipocyte interaction at the invasion front, shows low interobserver variability and can be assessed quickly and easily on routine H&E slides without any delay in turnaround time. We have shown previously that SARIFA-positivity is likely the morphologic correlate of an underlying distinct tumor biology,^13,14,17^ characterized by a broad dysregulation of RNA expression exhibiting a partial overlap with CMS1 (microsatellite instability immune subtype) and CMS4 (mesenchymal subtype).^14^ As CMS1 is characterized by an impaired DNA mismatch repair (MMR) system as well as a high *BRAF* mutation rate,^19^ and CMS3 is﻿ characterized by an overrepresentation of *K*﻿*RAS* mutations,^19^ we hypothesized that SARIFA may be related to these clinically used molecular alterations.

As MSI, *BRAF*, and *KRAS* testing are commonly performed in routine diagnostics nowadays for CRCs and as mutations in both genes are associated with poorer patient outcomes,^20^ studying the prognostic value of the ﻿SARIFA-status in these molecular subgroups is important to decide whether﻿ assessment of the SARIFA-status can lead to a further patient stratification in routine pathology. This is ofparticularl importance﻿ in the context of identifying those CRC﻿ patients who may ﻿benefit the most from adjuvant chemotherapy after surgery, which is still an unmet clinical need.^21^

Hence, the aim of our study was to investigate (1) whether SARIFA-status (SARIFA-positive vs. SARIFA-﻿negative) is related to *BRAF*, *RAS*, or MMR status, (2) whether SARIFA-status provides any additional prognostic information within molecular subgroups based on *BRAF*, *RAS*, and MMR status, and (3) whether the﻿ survival benefit from adjuvant therapy versus surgery-only differs according to SARIFA-status.

## METHODS

### Design and Study Population

This population-based series of colorectal cancer (CRC) patients was derived from the Netherlands Cohort Study (NLCS), a prospective cohort study that has been described in detail previously.^22^ Initiated in September 1986, the NLCS included 120,852 individuals aged 55 to 69 years. At baseline, participants completed a mailed, self-administered questionnaire on diet and other cancer risk factors.^22^ By completing and returning the questionnaire, participants agreed to participate in the study.

The NLCS was approved by the institutional review boards of the TNO Quality of Life Research Institute (Zeist, the Netherlands) and Maastricht University (Maastricht, the Netherlands). In addition, ethical approval was obtained from the Medical Ethical Committee (METC) of Maastricht University Medical Center+ (MUMC+).

**FIGURE 1:**
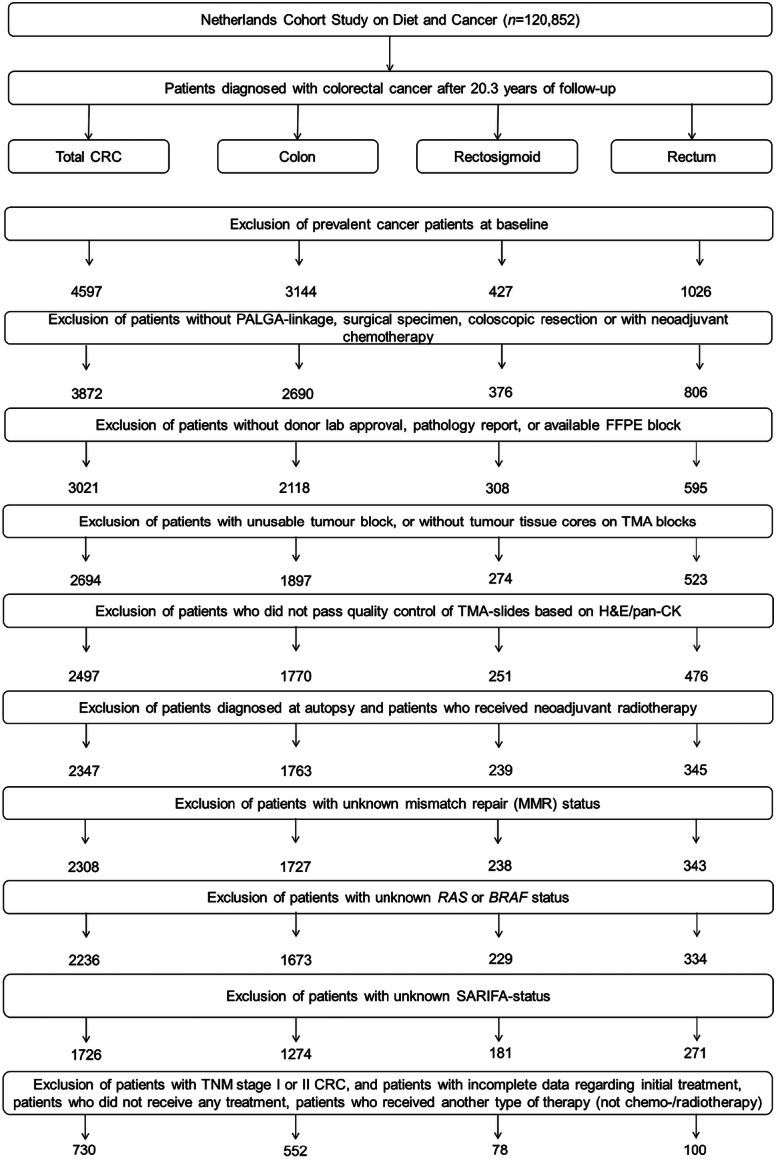
Flow diagram of the number of colorectal cancer patients available for analyses in the Netherlands Cohort Study (NLCS), 1986–2006. CRC indicates colorectal cancer; PALGA, Netherlands pathology database; TMA, tissue microarray.

6pt?>Cancer incidence follow-up was established annually through linkage with the Netherlands Cancer Registry and PALGA, the national Dutch Pathology Registry, covering 20.3 years of follow-up (September 17, 1986, until January 1, 2007).^23,24^ The estimated completeness of this follow-up exceeded 96%.^25^ After excluding individuals with a prior history of cancer (excluding non-melanoma skin cancer) at baseline, the study included 4597 incident CRC patients (Fig. 1).

### Tissue Collection and TMA Construction

From 2012 to 2017, formalin-fixed paraffin-embedded (FFPE) tissue blocks from CRCs were collected as part of the Rainbow-Tissue MicroArray (TMA) project.^26^ Details on TMA construction have been described previously.^27^ In total, 78 TMA blocks were constructed containing three 0.6 mm cores from the tumor and 3 from the normal epithelium of 2694 CRC patients (Fig. 1). In addition, for a previous study,^28^ two 20μm tissue sections had been cut from the tumor FFPE blocks for DNA extraction.

### Immunohistochemical Assessment of MMR Status

MMR status, as a proxy for microsatellite instability (MSI),^29^ was determined as part of a previous study.^27^ In short, 5μm thick serial TMA sections were subjected to immunohistochemistry (IHC) for MMR-related proteins (MLH1 and MSH2).^27^ Tumors with loss of either MLH1 or MSH2 expression, in the presence of internal positive controls, were categorized as ﻿MMR deficient (dMMR).^27^ For the current study, MMR status was known for 2308 CRC patients.

### DNA Isolation and Mutational Status

For a previous study,^28^ two 20μm thick FFPE tissue sections underwent manual deparaffinization, and DNA was isolated using the QIAsymphony (Qiagen) instrument, following the manufacturer’s protocol. Mutation analysis, targeting 32 mutations across 6 genes commonly mutated in CRC (ie, the ColoCarta Panel: *KRAS, NRAS, HRAS, BRAF, PIK3CA*, and *MET*), was previously conducted at the Institute for Immunology and Genetics (Kaiserslautern, Germany) using Matrix Assisted Laser Desorption Ionization Time of Flight (MALDI-TOF) mass spectrometry.^28^ In a previous study,^28^ patients testing positive for any mutation-specific assay were classified as mutant (mut) for the respective gene; patients with no detectable mutations were classified as wild-type (wt) for the respective gene; and patients for whom testing failed or for whom equivocal results were obtained (ie, one or more mutation-specific assay(s) failed and for other mutation-specific assays no detectable mutations were identified) were classified as having an unknown mutation status for the respective gene.^28^ For the current study, *KRAS*, *NRAS*, and *HRAS* were examined together as *RAS* mutational status.

After excluding patients with unknown mutational status for *KRAS, NRAS, HRAS*, or *BRAF* (*n*=72), 2236 patients were available for analyses (Fig. 1).

### Histopathological Assessment of SARIFA-status

SARIFA-status (positive vs. negative vs. unknown﻿) was established on digitized H&E-stained whole slide images (WSI) in line with our previous publications on SARIFA in CRC.^14,16,17,30^ From all NLCS CRC resection specimens, one﻿ single representative tumor containing H&E-stained tissue section (with deepest invasion) had been previously scanned at ×40 magnification (Aperio XT whole slide scanner, Aperio TechnologiesA), and digital slides were accessed by using QuPath (https://qupath.github.io/).^30,31^ We have previously already shown that this selection criterion is reliable.^16,30^

Cases were deemed not ﻿assessable (SARIFA-unknown) if scans of the initial H&E-stained whole sections of the cohort were unavailable for review (n=30) or because of other assessment-related issues (n=490; particularly if only superficial tumor parts were present e.g. absence of the tumor-fat interface). SARIFA-positivity was defined as a direct tumor-adipocyte interaction at the invasion front; a direct tumor-adipocyte contact of at least one﻿ tumor gland or at least a group of five or ﻿more tumor cells without intervening inflammatory infiltrate or (desmoplastic) stroma was required. The presence of one such area was sufficient to categorize the whole case as SARIFA-positive. Otherwise, the case was classified as SARIFA-negative. All CRCs were classified by J.Z. and/or N.G.R., supervised by B.M. and H.I.G., both senior board-certified pathologists. We have already proven that interobserver variability for assessment of the ﻿SARIFA-status in CRC is low.^16^ Please refer also to our previous publication on SARIFA-status in the NLCS for further information.^30^

**FIGURE 2:**
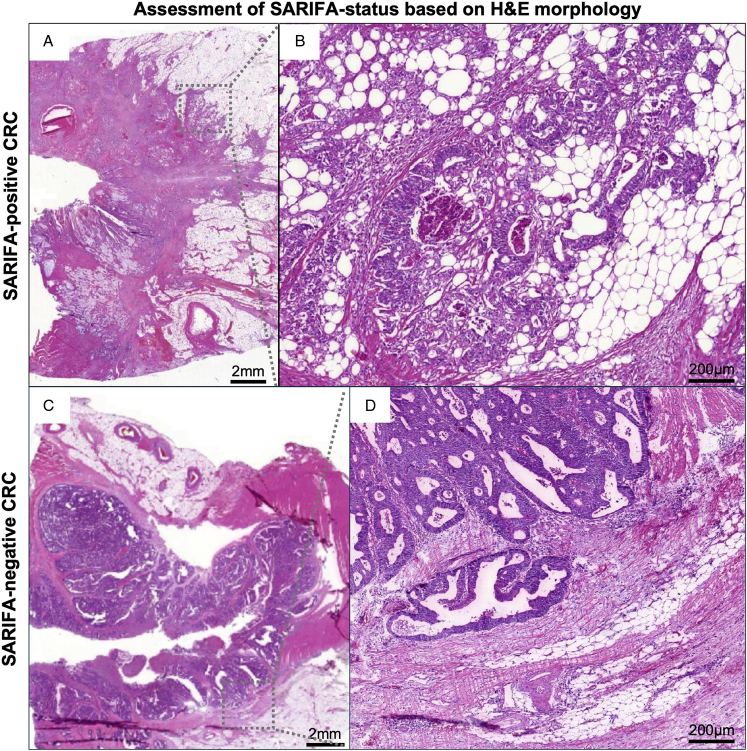
H&E-based histopathological﻿ assessment of SARIFA-status. SARIFA-positivity is defined as direct contact between at least 1﻿ tumor gland or a group of at least 5﻿ tumor cells and at least 1﻿ adipocyte at the invasion front (A, B). SARIFA-positivity is typically not seen in CRCs with a stromal or inflammatory reaction at the invasion front (C, D). A single focus of SARIFA-positivity is sufficient to classify a cancer as SARIFA-positive. CRC indicates colorectal cancer; H&E, hematoxylin-and-eosin; SARIFA, Stroma AReactive Invasion Front Area.

Examples of SARIFA-positive and SARIFA-negative CRC within the NLCS are displayed in Figure 2 and Supplementary Figures S1 and S2, Supplemental Digital Content 1, http://links.lww.com/PAS/C100.

### Clinical Characteristics and Follow-up

Follow-up for the vital status of the CRC patients was conducted through linkage to the Central Bureau of Genealogy and municipal population registries until December 31, 2012. Patients diagnosed with CRC at autopsy (n=5) and patients who received neoadjuvant radiotherapy (n=145) were excluded from analyses (Fig. 1).

Cause of death was obtained from Statistics Netherlands. CRC-specific deaths included those with an underlying cause attributed to malignant neoplasms of the colon, rectosigmoid junction, or rectum. Vital status was available for 2235 (>99%) patients, and CRC-specific death for 2200 (98.4%) patients.

Information on patient and tumor characteristics, including age at diagnosis, pathologic tumor-node-metastasis (pTNM) stage, tumor location, differentiation grade, and primary adjuvant therapy (i.e. treatments included in the initial treatment plan drawn up after diagnosis), were retrieved from the cancer registry or PALGA histopathology records. For the analysis of survival benefits from adjuvant therapy versus surgery-only according to SARIFA-status, patients with unknown SARIFA-status (n=510), patients with no indication for adjucant chemotheray (﻿pTNM stage I (n=312) or stage II (n=661) CRC), as well as patients with incomplete data regarding initial treatment (n=16), patients who did not receive any treatment (n=5), or patients who received another type of therapy (n=2) were initially ﻿excluded leaving 730 CRC patients for this subanalysis (Fig. 1). However, pTNM stage II patients were later re-included for exploratory subgroup analyses, as certain high-risk features may still warrant adjuvant treatment in this group.﻿

### Statistical Analyses

Descriptive statistics and frequency distributions were computed for clinical and molecular characteristics. Variations between SARIFA-positive and SARIFA-negative patient subgroups were assessed using χ^2^ tests for categorical variables and Kruskal-Wallis tests for continuous variables. The primary outcome measures of this study were CRC-specific survival, defined as the duration from CRC diagnosis to CRC-related death or end of follow-up, and overall survival, defined as the duration from CRC diagnosis to death from any cause or end of follow-up. Due to the limited number of events in the follow-up period exceeding 10 years (CRC-specific deaths: n=33, 3.4%; overall deaths: n=264, 15.3%), all survival analyses were restricted to 10 years of follow-up.

The relationship between (1) SARIFA-status and CRC-specific and overall survival within molecular subgroups based on *BRAF*, *RAS*, and MMR status, as well as (2) the relationship between SARIFA-status and CRC-specific and overall survival benefit from adjuvant therapy versus surgery-only﻿, was examined using Kaplan-Meier curves and Wilcoxon tests. In addition, hazard ratios (HRs) and 95% CIs were estimated using Cox proportional hazards regression analyses. The statistical significance of the interaction between SARIFA-status and therapeutic intervention was assessed using likelihood ratio tests comparing the multivariable-adjusted models with and without the interaction term.

The proportional hazards assumption was tested using the scaled Schoenfeld residuals^32^ by evaluating log-transformed survival curves or by introducing time-covariate interactions into the models. HRs were adjusted for a set of a priori selected prognostic factors,^27^ including age at diagnosis (years), sex (men/women), tumor location (colon, rectosigmoid, and rectum), pTNM stage (I, II, III, and IV), grade of differentiation (well, moderate, and poor/undifferentiated), and adjuvant therapy (no and yes). A separate category (“unknown”) was used for patients with unknown clinical information on pTNM stage, differentiation grade, or adjuvant therapy to enable the inclusion of these patients in the Cox proportional hazards models.

The disease stage was determined using the pTNM classification based on the edition valid at the time of cancer diagnosis, resulting in the use of 5 different TNM editions (UICC TNM edition 3-6).^27^ However, it is worth noting that the primary TNM stage categories (I/II/III/IV) remained essentially unchanged over the years.^33^ Year of diagnosis and the pTNM version were considered as potential confounders and only retained in the final models if they introduced a ≥10% change in HRs.

All analyses were conducted using Stata Statistical Software: Release 16 (StataCorp.). *P* values <0.05 were considered statistically significant.

## RESULTS

After excluding patients with unknown mismatch repair (MMR) status (n=39) or unknown *RAS* or *BRAF* mutational status (n=72), 2236 colorectal cancer (CRC) patients were available for analyses (Fig. 1). In total, 1228 (55.7%) patients were classified as SARIFA-negative, 498 (22.6%) as SARIFA-positive, and 510 (22.8%) as SARIFA-unknown ( see the Methods section). The frequency of SARIFA-positive CRCs among all classified cases was 28.9%.

### Clinical Characteristics

Clinical characteristics of the total series of incident CRC patients with known SARIFA-status within the Netherlands Cohort Study (NLCS), as well as according to SARIFA-status (positive vs. negative) have been previously published.^30^ The clinicopathologic characteristics of the patients included in the current study (also refer to flow diagram in Fig. 1) are shown in Supplementary Table S1, Supplemental Digital Content 2, http://links.lww.com/PAS/C101. Briefly, SARIFA-positive and SARIFA-negative patients differed significantly regarding tumor location, pTNM stage, depth of ﻿tumor invasion (pT), lymph node status (pN), differentiation grade, and adjuvant therapy. SARIFA-positivity was associated with several adverse clinicopathologic risk factors: an advanced pTNM stage, increased pT category﻿, increased pN category﻿, and poorly/undifferentiated cancers. Accordingly, patients with SARIFA-positive CRC more frequently received adjuvant therapy compared with patients with SARIFA-negative CRC (22.7% vs. 14.3%, respectively).

### Relationship Between SARIFA-status, *BRAF* Mutation Status, *RAS* Mutation Status, and Mismatch Repair Status

**FIGURE 3:**
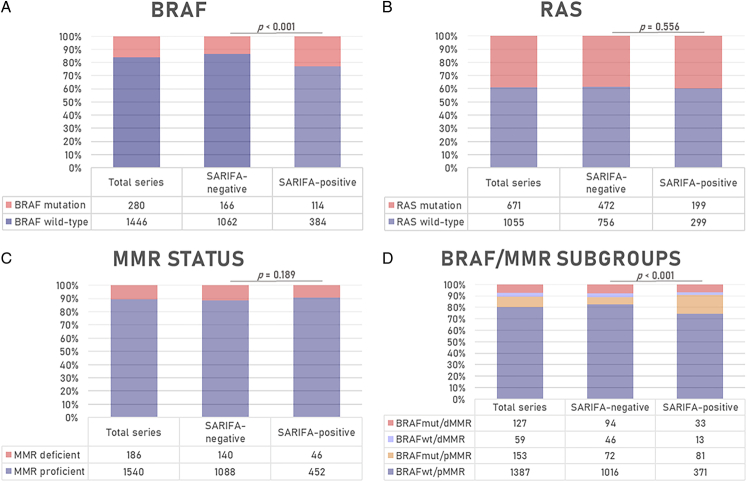
Relationship between SARIFA-status and molecular characteristics of colorectal cancer (n=1726) within the Netherlands Cohort Study (NLCS; 1986–2006): (A) *BRAF* mutational status, (B) *RAS* mutational status, (C) *MMR* status, and (D) *BRAF*/MMR subgroups. *P* value for the χ^2^ test. The *RAS* group comprises all CRCs﻿with mutations in *KRAS* and/or *NRAS*. *HRAS* mutations were not observed. *BRAF* indicates V-Raf Murine Sarcoma Viral Oncogene Homolog B; *MMR*, mismatch repair; *RAS*, Rat sarcoma.

Mutations in *BRAF* or *RAS* were observed in 280 (16.2%) and 671 (38.9%) CRCs with known SARIFA-status, respectively. MMR deficiency (dMMR) was found in 186 (10.8%) CRCs with known SARIFA-status. The relationship between SARIFA-status (positive vs. negative) and *BRAF*, *RAS*, and MMR status is shown in Figure 3. SARIFA-positive CRCs more frequently had *BRAF* mutations compared with SARIFA-negative CRCs (22.9% vs. 13.5%, *P*<0.001). No relationship was observed between SARIFA-status and *RAS* mutation status (*P*=0.556) or MMR status (*P*=0.189). Furthermore, SARIFA-positive CRCs more often were *BRAF*_mut_/pMMR compared with SARIFA-negative CRCs (16.3% vs. 5.9%, *P*<0.001).

### SARIFA-status and Survival Within Molecular Subgroups Based on *BRAF*, *RAS*, and MMR Status

The median (range) follow-up time since diagnosis was 4.8 years (0.0027 to 25.99y). Survival analyses were restricted to 10 years of follow-up. During these first 10 years of follow-up, 1458 deaths were observed, of which 927 (63.6%) were CRC-related deaths. We have previously shown in multivariable-adjusted analysis that SARIFA-positivity is associated with a significantly poorer CRC-specific and overall survival, independent of several clinically known risk factors (especially irrespective of pTNM stage).^30^

**FIGURE 4:**
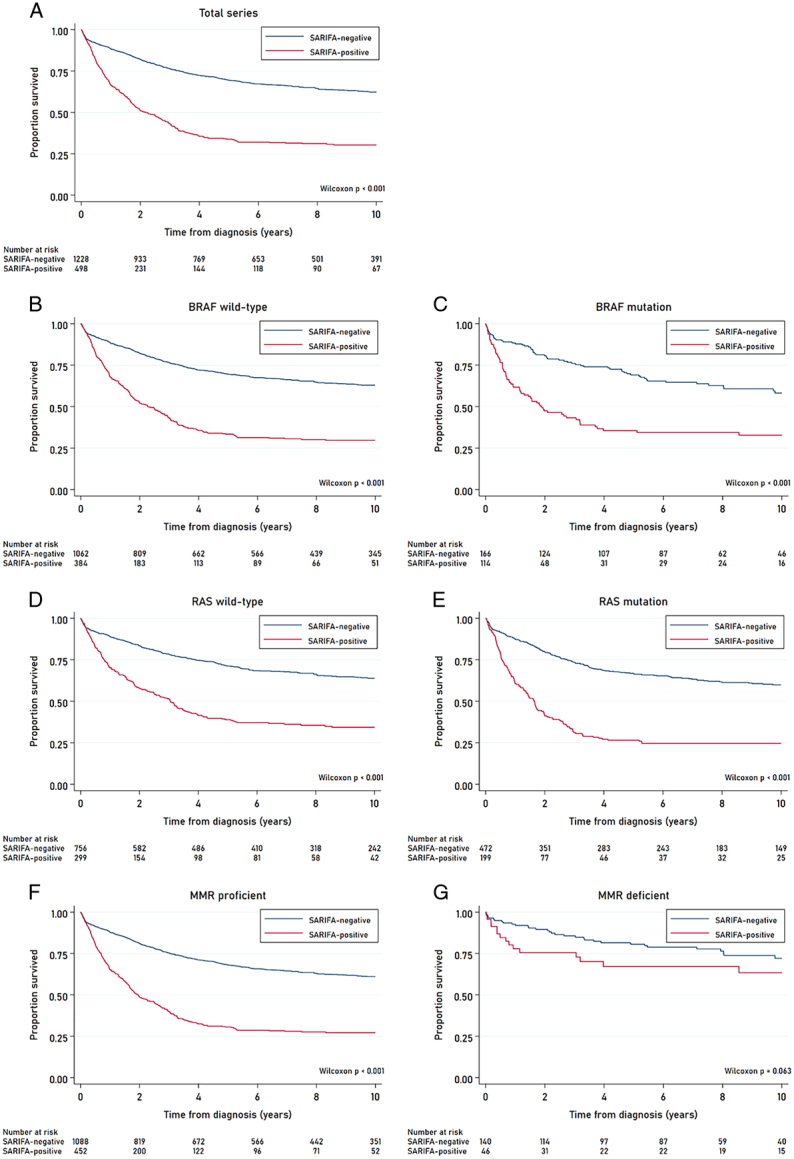
Univariable Kaplan-Meier curves showing the CRC-specific survival of patients within the Netherlands Cohort Study (NLCS; 1986–2006) according to SARIFA-status for (A) the total series of CRC patients, as well as within molecular subgroups based on *BRAF*, *RAS*, and MMR status: (B) *BRAF*_wt_, (C) *BRAF*_mut_, (D) *RAS*_wt_, (E) *RAS*_mut_, (F) pMMR, and (G) dMMR. *BRAF* indicates V-Raf Murine Sarcoma Viral Oncogene Homolog B; ​​​​​​*CHT*, chemotherapy; *MMR*, mismatch repair; *RAS*, Rat sarcoma; *RT*, radiotherapy; *SARIFA*, Stroma AReactive Invasion Front Areas.

In our current study, univariable Kaplan-Meier curves showed that CRC-specific and overall survival differed significantly between patients with ﻿SARIFA-positive and SARIFA-negative CRCs across all molecular subgroups based on *BRAF*, *RAS*, and MMR status, except for the dMMR subgroup (Fig. 4 and Supplementary Figure S3, Supplemental Digital Content 1, http://links.lww.com/PAS/C100). Patients with SARIFA-positive CRC had significantly poorer CRC-specific and overall survival compared with patients with SARIFA-negative CRC regardless of *BRAF* or *RAS* mutational status (Table 1). Associations between SARIFA-status and CRC-specific and overall survival failed to reach statistical significance within the dMMR﻿ subgroup (*P*_CRC-specific_=0.063 and *P*_overall_=0.161; Fig. 4 and Supplementary Figure S3, Supplemental Digital Content 1, http://links.lww.com/PAS/C100), most likely due to low statistical ﻿power of the analysis due to the limited number of patients within this subgroup, and the already known favorable prognosis of patients with ﻿dMMR CRC in general^34^ (Table 1).

**TABLE 1:** Univariable and Multivariable-adjusted Hazard Ratios for Associations Between SARIFA-status and Survival of Colorectal Cancer ﻿Patients Within the Netherlands Cohort Study (NLCS, 1986–2006) within Molecular Subgroups (*BRAF*_wt_/*BRAF*_mut_, *RAS*_wt_/*RAS*_mut_, pMMR/dMMR; n=2236)

		CRC-specific survival			Overall survival		
			HR (95% CI)			HR (95% CI)	
	N	CRC deaths (%)	Univariable	Multivariable-adjusted*	Deaths (%)	Univariable	Multivariable-adjusted*
Overall							
SARIFA-negative	1228	411 (33.5)	1.00 (Reference)	1.00 (Reference)	729 (59.4)	1.00 (Reference)	1.00 (Reference)
SARIFA-positive	498	319 (64.1)	2.76 (2.38-3.20)	1.59 (1.35-1.87)	405 (81.3)	2.11 (1.87-2.39)	1.42 (1.24-1.63)
SARIFA-unknown	510	197 (38.6)	1.28 (1.08-1.52)	1.21 (1.01-1.45)	324 (63.5)	1.20 (1.05-1.36)	1.15 (1.00-1.32)
*BRAF*							
Wild-type							
SARIFA-negative	1062	351 (33.1)	1.00 (Reference)	1.00 (Reference)	630 (59.3)	1.00 (Reference)	1.00 (Reference)
SARIFA-positive	384	249 (64.8)	2.82 (2.40-3.32)	1.55 (1.29-1.86)	315 (82.0)	2.14 (1.87-2.45)	1.35 (1.16-1.58)
SARIFA-unknown	432	162 (37.5)	1.24 (1.03-1.49)	1.16 (0.95-1.42)	268 (62.0)	1.15 (0.99-1.32)	1.09 (0.94-1.27)
Mutation							
SARIFA-negative	166	60 (36.1)	1.00 (Reference)	1.00 (Reference)	99 (59.6)	1.00 (Reference)	1.00 (Reference)
SARIFA-positive	114	70 (61.4)	2.42 (1.71-3.42)	1.68 (1.14-2.49)	90 (78.9)	1.99 (1.50-2.66)	1.70 (1.23-2.36)
SARIFA-unknown	78	35 (44.9)	1.51 (1.00-2.30)	1.78 (1.12-2.83)	56 (71.8)	1.49 (1.07-2.07)	1.74 (1.21-2.94)
*RAS*							
Wild-type							
SARIFA-negative	756	241 (31.9)	1.00 (Reference)	1.00 (Reference)	446 (59.0)	1.00 (Reference)	1.00 (Reference)
SARIFA-positive	299	176 (58.9)	2.55 (2.10-3.10)	1.47 (1.18-1.83)	238 (79.6)	2.00 (1.71-2.35)	1.46 (1.22-1.74)
SARIFA-unknown	307	108 (35.2)	1.19 (0.95-1.50)	1.02 (0.80-1.30)	192 (62.5)	1.15 (0.97-1.37)	1.04 (0.86-1.25)
Mutation							
SARIFA-negative	472	170 (36.0)	1.00 (Reference)	1.00 (Reference)	283 (60.0)	1.00 (Reference)	1.00 (Reference)
SARIFA-positive	199	143 (71.9)	3.10 (2.47-3.88)	1.78 (1.38-2.29)	167 (83.9)	2.31 (1.90-2.80)	1.39 (1.11-1.73)
SARIFA-unknown	203	89 (43.8)	1.41 (1.09-1.82)	1.56 (1.19-2.05)	132 (65.0)	1.26 (1.03-1.55)	1.39 (1.11-1.73)
Mismatch repair (MMR) status							
Proficient (pMMR)							
SARIFA-negative	1088	379 (34.8)	1.00 (Reference)	1.00 (Reference)	651 (59.8)	1.00 (Reference)	1.00 (Reference)
SARIFA-positive	452	304 (67.3)	2.87 (2.46-3.34)	1.60 (1.35-1.90)	378 (83.6)	2.23 (1.96-2.54)	1.43 (1.24-1.65)
SARIFA-unknown	444	177 (39.9)	1.26 (1.06-1.51)	1.19 (0.99-1.44)	281 (63.3)	1.17 (1.02-1.35)	1.13 (0.97-1.31)
Deficient (dMMR)							
SARIFA-negative	140	32 (22.9)	1.00 (Reference)	1.00 (Reference)	78 (55.7)	1.00 (Reference)	1.00 (Reference)
SARIFA-positive	46	15 (32.6)	1.62 (0.88-2.99)	1.41 (0.71-2.82)	27 (58.7)	1.21 (0.78-1.87)	1.30 (0.80-2.12)
SARIFA-unknown	66	20 (30.3)	1.52 (0.87-2.66)	1.53 (0.82-2.88)	43 (65.2)	1.39 (0.96-2.02)	1.38 (0.92-2.08)

* Adjusted for age at diagnosis (years), sex (male, female), tumor location (colon, rectosigmoid, rectum), pTNM stage (I, II, III, IV, unknown), differentiation grade (well, moderate, poor/undifferentiated, unknown), adjuvant therapy (no, yes, unknown), and MMR status (proficient, deficient).

*BRAF* indicates V-Raf Murine Sarcoma Viral Oncogene Homolog B; CRC, colorectal cancer; HR, hazard ratio; *RAS*, Rat sarcoma; SARIFA, Stroma AReactive Invasion Front Areas.

In multivariable-adjusted Cox regression models, SARIFA-positivity remained a significant predictor of CRC-specific and overall survival regardless of *BRAF* or *RAS* mutational status (Table 1). Observed associations between SARIFA-positivity and CRC-specific as well as overall survival were stronger in the *BRAF* mutation subgroup compared with the *BRAF* wild-type subgroup (CRC-specific: HR_BRAF,mut_: 1.68; 95% CI: 1.14-2.49 vs. HR_BRAF,wt_: 1.55; 95% CI: 1.29-1.86; overall: HR_BRAF,mut_: 1.70; 95% CI: 1.23-2.36 vs. HR_BRAF,wt_: 1.35; 95% CI: 1.16-1.58; Table 1). Furthermore, associations between SARIFA-positivity and CRC-specific survival were stronger in the *RAS* mutation subgroup compared with the *RAS* wild-type subgroup (CRC-specific: HR_RAS,mut_: 1.78; 95% CI: 1.38-2.29 vs. HR_RAS,wt_: 1.47; 95% CI: 1.18-1.83; Table 1). Within the pMMR subgroup, SARIFA-positivity was associated with a significantly worse CRC-specific and overall survival (HR_CRC-specific_: 1.60; 95% CI: 1.35-1.90 and HR_overall_: 1.43; 95% CI: 1.24-1.65; Table 1).

When analysing the prognostically relevant combinational subgroups based on both *BRAF* mutational status and MMR status, we observed significant differences in CRC-specific and overall survival according to SARIFA-status within the﻿ *BRAF*_wt_/pMMR, *BRAF*_mut_/pMMR, and *BRAF*_mut_/dMMR subgroups, but not the *BRAF*_wt_/dMMR subgroup (Supplementary Figures S4 and S5, Supplemental Digital Content 1, http://links.lww.com/PAS/C100). In multivariable-adjusted analyses, SARIFA-positivity was associated with poorer CRC-specific and overall survival within the *BRAF*_wt_/pMMR (HR_CRC-specific_: 1.67; 95% CI: 1.40-1.98 and HR_overall_: 1.47; 95% CI: 1.27-1.70) and *BRAF*_mut_/pMMR (HR_CRC-specific_: 1.83; 95% CI: 1.15-2.91 and HR_overall_: 2.03; 95% CI: 1.34-3.08) subgroups (Supplementary Table S2, Supplemental Digital Content 3, http://links.lww.com/PAS/C102).

Additional analyses restricted to patients with ﻿locally advanced pT3 or pT4 CRC showed that SARIFA-positivity remained an independent prognostic factor (pT3: HR_CRC-specific_: 1.49; 95% CI: 1.24-1.78 and HR_overall_: 1.36; 95% CI: 1.17-1.58; pT4: HR_CRC-specific_: 1.79; 95% CI: 1.19-2.69 and HR_overall_: 1.74; 95% CI: 1.21-2.50; Supplementary Table S3, Supplemental Digital Content 4, http://links.lww.com/PAS/C103). Furthermore, SARIFA-positivity retained its independent prognostic value within molecular subgroups (Supplementary Table S4, Supplemental Digital Content 5, http://links.lww.com/PAS/C104: *BRAF, RAS*, pMMR; Supplementary Table S5, Supplemental Digital Content 6, http://links.lww.com/PAS/C105: BRAF/MMR subgroups) when analyses were restricted to pT3/pT4 CRCs only. The results of these additional analyses were consistent with those of our main analyses presented in Table 1.

### SARIFA-status and Survival Benefit From Adjuvant Therapy

After excluding patients with unknown SARIFA-status (n=510), pTNM stage I (n=312) or stage II CRC (n=661), and patients with incomplete data regarding initial treatment (n=16), patients who did not receive any treatment (n=5), or patients who received another type of therapy (n=8), 730 CRC patients were available for analyses (Fig. 1).

Univariable Kaplan-Meier curves showed significant differences in CRC-specific and overall survival across therapeutic intervention groups for the total series of CRC patients, as well as for patients with ﻿SARIFA-positive orSARIFA-negative CRC﻿ (Supplementary Figures S6 and S7, Supplemental Digital Content 1, http://links.lww.com/PAS/C100). In general, CRC patients who received surgery only had a poorer CRC-specific and overall survival compared with CRC patients who received surgery plus adjuvant therapy.

Multivariable-adjusted analyses showed that for the total series of pTNM stage III-IV CRC patients, patients who received adjuvant (chemo)therapy had a significantly better CRC-specific (HR: 0.71; 95% CI: 0.58-0.87) and overall survival (HR: 0.68; 95% CI: 0.56-0.82) compared with patients who received surgery only (Table 2). Within the subgroup of patients with SARIFA-positive CRC, patients who received surgery plus adjuvant (chemo)therapy showed a significantly improved CRC-specific (HR: 0.59; 95% CI: 0.44-0.79) and overall survival (HR: 0.60; 95% CI: 0.46-0.78) compared with patients who received surgery only (Table 2). In contrast, within the subgroup of patients with ﻿SARIFA-negative CRC﻿, no significant CRC-specific survival benefit was observed for surgery plus adjuvant (chemo)therapy versus surgery only (HR: 0.81; 95% CI: 0.59-1.09), while a significant overall survival benefit was observed (HR: 0.72; 95% CI: 0.55-0.95; Table 2).

**TABLE 2:** Association Between Adjuvant Therapy and CRC-specific and Overall Survival of pTNM Stage III and IV Colorectal Cancer Patients ﻿Within the Netherlands Cohort Study (NLCS, 1986–2006), According to SARIFA-status (SARIFA-positive and SARIFA-negative; n=730)

		CRC-specific survival			Overall survival		
			HR (95% CI)			HR (95% CI)	
	N	CRC deaths (%)	Univariable	Multivariable-adjusted*	Deaths (%)	Univariable	Multivariable-adjusted*
Colorectal cancer							
Surgery only	504	339 (67.3)	1.00 (Reference)	1.00 (Reference)	425 (84.3)	1.00 (Reference)	1.00 (Reference)
Surgery + adjuvant therapy	226	148 (65.5)	0.73 (0.60-0.88)	0.71 (0.58-0.87)	175 (77.4)	0.68 (0.57-0.81)	0.68 (0.56-0.82)
*Surgery + adjuvant CHT*	*189*	*126* (*66.7)*	*0.76 (0.62-0.93)*	*0.69 (0.55-0.85)*	*147* (*77.8)*	*0.70 (0.58-0.84)*	*0.65 (0.54-0.80)*
*Surgery + adjuvant RT*	*37*	*22* (*59.5)*	*0.60 (0.39-0.92)*	*0.92 (0.56-1.51)*	*28* (*75.7)*	*0.60 (0.41-0.87)*	*0.91 (0.58-1.42)*
SARIFA-positive							
Surgery only	231	174 (75.3)	1.00 (Reference)	1.00 (Reference)	208 (90.0)	1.00 (Reference)	1.00 (Reference)
Surgery + adjuvant therapy	100	76 (76.0)	0.72 (0.55-0.94)	0.59 (0.44-0.79)	88 (88.0)	0.70 (0.55-0.90)	0.60 (0.46-0.78)
*Surgery + adjuvant CHT*	*90*	*70* (*77.8)*	*0.75 (0.57-0.99)*	*0.59 (0.44-0.80)*	*80* (*88.9)*	*0.72 (0.56-0.93)*	*0.59 (0.45-0.78)*
*Surgery + adjuvant RT*	*10*	*6* (*60.0)*	*0.49 (0.22-1.11)*	*0.55 (0.21-1.46)*	*8* (*80.0)*	*0.54 (0.27-1.09)*	*0.65 (0.28-1.50)*
SARIFA-negative							
Surgery only	273	165 (60.4)	1.00 (Reference)	1.00 (Reference)	217 (79.5)	1.00 (Reference)	1.00 (Reference)
Surgery + adjuvant therapy	126	72 (57.1)	0.72 (0.54-0.95)	0.81 (0.59-1.09)	87 (69.0)	0.65 (0.51-0.83)	0.72 (0.55-0.95)
*Surgery + adjuvant CHT*	*99*	*56* (*56.6)*	*0.71 (0.52-0.96)*	*0.74 (0.53-1.04)*	*67* (*67.7)*	*0.64 (0.48-0.84)*	*0.66 (0.49-0.89)*
*Surgery + adjuvant RT*	*27*	*16* (*59.3)*	*0.74 (0.44-1.24)*	*1.20 (0.66-2.17)*	*20* (*74.1)*	*0.69 (0.44-1.10)*	*1.07 (0.63-1.83)*

* Adjusted for age at diagnosis (years), sex (male, female), tumor location (colon, rectosigmoid, rectum), pTNM stage (III, IV, unknown), differentiation grade (well, moderate, poor/undifferentiated, unknown), and MMR status (proficient, deficient).

CHT indicates chemotherapy; CRC, colorectal cancer; HR, hazard ratio; RT, radiotherapy; SARIFA, Stroma AReactive Invasion Front Areas.

There was no significant interaction between SARIFA-status and adjuvant therapy for CRC-specific survival (*P*_likelihood_=0.30) or overall survival (*P*_likelihood_=0.55). When adding pTNM stage II CRCs to the cohort (stages II-IV, n=1385), similar associations were observed (interaction for CRC-specific survival: *P*_likelihood_=0.45, for overall survival: *P*_likelihood_=0.09; Supplementary Table S6, Supplemental Digital Content 7, http://links.lww.com/PAS/C106). However, no significant overall survival benefit from adjuvant therapy was observed in the subgroup of patients with ﻿SARIFA-negative CRC (HR: 0.82; 95% CI: 0.65-1.04; Supplementary Table S6, Supplemental Digital Content 7, http://links.lww.com/PAS/C106), which raises the question whether﻿ patients with ﻿SARIFA-negative CRC do truly benefit from adjuvant therapy.

To determine whether SARIFA-status merely serves as a proxy for the extent of pericolonic adipose tissue involvement, Cox regression analyses were repeated, focusing solely on advanced pT3 and pT4 tumors. The results (Supplementary Table S7, Supplemental Digital Content 8, http://links.lww.com/PAS/C107 and Supplementary Table S8, Supplemental Digital Content 9, http://links.lww.com/PAS/C108) were consistent with those from the main analyses, which included all pT categories﻿, showing that patients with pT3/pT4 CRC had a cancer-specific as well as overall survival benefit from adjuvant therapy whereas patients with ﻿SARIFA-negative CRC did not. Furthermore, analyses were repeated, focusing exclusively on pTNM stage II colon cancer/CRC, as identifying which stage II patients derive the most survival benefit from adjuvant therapy remains an ongoing challenge. However, due to the limited number of patients in this subgroup, no significant associations were observed (Supplementary Table S9, Supplemental Digital Content 10, http://links.lww.com/PAS/C109 and Supplementary Table S10, Supplemental Digital Content 11, http://links.lww.com/PAS/C110).

## DISCUSSION

In this large population-based series of colorectal cancers (CRC), in which we previously validated the independent negative prognostic value of our H&E-based biomarker SARIFA (Stroma AReactive Invasion Front Areas),^30^ we now investigated whether the strong prognostic value of SARIFA-status remains within molecular subgroups based on *BRAF*, *RAS*, and MMR status and whether﻿ SARIFA-positivity is associated with a differential response to adjuvant therapy. Moreover, we studied the relationship between *BRAF*, *KRAS* and/or MMR-status, and SARIFA-status.

Whereas we could not find any SARIFA-status ﻿dependent changes at a genetic level in our previous studies on CRC,^14,17^ which were based on small to moderate cohort sizes (n=45 and n=207), we now observed a significantly higher frequency of *BRAF* mutations within SARIFA-positive CRCs compared with SARIFA-negative CRCs. This finding is in line with the fact that we have already observed higher numbers of *BRAF* mutations within Warburg-high CRCs^28^ as well as an association between SARIFA-positivity and the Warburg-high subtype.^30^ In addition, we have already seen a higher number of harmful *BRAF V600E* mutations in SARIFA-positive cases without reaching statistical significance (TCGA-COAD and TCGA-READ:^35^ SARIFA-positive 10.4% vs. 8.0% SARIFA-negative, *P* = 0.27^14^). Interestingly, we could also observe an enrichment of *BRAF*-mutant pMMR﻿ CRCs within SARIFA-positive CRC﻿. As *BRAF*-mutant pMMR CRCs are known to show a particularly aggressive behavior with reduced survival outcomes,^36,37^ these findings underscore that SARIFA-positivity is linked to an aggressive tumor biology. The prognostic relevance of tumor-adipocyte interaction has also been demonstrated by several studies deploying deep-learning algorithms on H&E slides.^38–40^ The large number of patients in the current cohort enabled us to link a specific genotype to SARIFA-positivity for the first time. Nevertheless, taking into account our current and prior findings,^14^ we still believe that SARIFA-positivity is not (or only to a small degree) genetically determined (after all, *BRAF* mutation frequency was only about 10% higher in SARIFA-positive than in SARIFA-negative CRCs﻿), but rather relies on the complex interplay of immune, stromal, and metabolic changes.^14^ We could already demonstrate that SARIFA-positivity, which is correlated to higher tumor budding,^16^ is also associated with CMS4, which is characterized by an upregulation of a stromal gene expression profile and presumably linked to epithelial-mesenchymal transition (EMT).^14^ Even though SARIFA-status and tumor budding (often considered as the histological phenotypeof EMT) are H&E-based biomarkers with relevant differences, these results indicate that they also show a biological overlap (enriched for CMS4 and *BRAF* mutated CRCs and poor prognosis).

As it is already known that pMMR/microsatellite stability﻿, *BRAF-*mutant, and *RAS-*mutant CRCs are associated with a poor prognosis,^20,41^ we investigated whetherSARIFA-positivity is associated with poor survival outcomes within molecular subgroups based on *BRAF*, *RAS*, and MMR status. SARIFA-positivity was indeed associated with a reduced CRC-specific and overall survival in almost all molecular subgroups. Even in MSI CRCs, which are known to show favorable outcomes independent of *KRAS* or *BRAF* status,^20^ SARIFA-positivity seemed to be associated with a worse CRC-specific (*P*=0.063) and reduced overall survival (*P*=0.161). Strikingly, SARIFA-status could also separate *BRAF-*mutant/microsatellite stability CRCs, which are, as already stated, considered high-risk CRCs with dismal prognosis.^37^ These results demonstrate for the first time that the SARIFA-status provides further patient stratification even within molecular subgroups based on *BRAF*, *RAS*, and MMR status, which are currently established for a relevant subset of CRCs in routine practice. Investigating whetherSARIFA-positivity retains its strong prognostic value also within CMS subgroups^42^ could be of further interest in future projects. In addition, we have previously shown that SARIFA-status seems to be superior to conventional prognostic clinicopathological﻿ and histological biomarkers in CRC, such as lymphovascular invasion, tumor-stroma-ratio, or grading.^14,43^ SARIFA-positivity may correlate positively with the extent/depth of pericolonic adipose tissue infiltration in pT3 CRCs, which is a known prognostic feature^44,45^; for our cohort, these data are not available and should be considered in further studies.

As identifying those CRC patients who benefit most from adjuvant therapy is still a pressing clinical need, and as we have already observed a SARIFA-dependent differential drug sensitivity^14^ by deploying *oncoPredict*, which is a computational tool to predict drug response from transcriptional data,^46^ we investigated the survival benefit from adjuvant therapy according to SARIFA-status. Our results here suggest that patients with ﻿SARIFA-positive CRC derive a CRC-specific survival benefit from adjuvant therapy, whereas this survival benefit was not observed for patients with SARIFA-negative CRC﻿. SARIFA-status assessment c﻿ould be implemented into routine pathologic reports easily as it is solely based on H&E histopathology and, therefore, would not be ﻿associated with additional costs (except for the pathologist’s time), and would not increase turnaround time. In future studies, SARIFA-positivity should be considered as an additional high-risk factor for patients with stage II CRCs,^47,48^ which could potentially trigger provision of ﻿adjuvant chemotherapy after surgery. Some studies have already shown that other histopathologic invasion front biomarkers, like tumor budding^49^ or desmoplastic reaction pattern,^50^ may potentially predict a survival benefit from adjuvant chemotherapy. However, compared with the assessment of SARIFA-status,^16^ these other 2﻿ histological﻿ biomarkers show a higher interobserver variability.^50,51^ Interestingly, intratumoral stroma content, which is higher in SARIFA-positive CRCs and associated with disease recurrence,^43^ failed to predict response to 5-fluorouracil in a post-hoc analysis of the QUASAR trial.^52^ Besides predicting response to conventional chemotherapy, SARIFA-status could potentially stratify patients for immunotherapyc^17^ and/or other ﻿novel treatment approaches, directly targeting the upregulated lipid metabolism in SARIFA-positive CRCs,^14^ for example, via CD36 or FABP4 inhibition.

However, the results of the current study should be interpreted cautiously for several reasons. First, with regard to overall survival, both, patients withSARIFA-positive as well as patients with SARIFA-negative CRCs benefitted from adjuvant therapy. Second, treatment interactions did not show statistical significance. Third, adjuvant therapy data did not contain exact therapy regimens (ie﻿ we did not have any detailed clinical information available regarding the dosage, duration, or exact type of treatment), and patients were not randomized to different treatment/observation arms as the NLCS was a population-based observational study. Therefore, post-hoc analysis or implementation of SARIFA-status assessment in prospective clinical trials should be performed.

For our cohort, we have previously established Warburg-subtyping by performing different immunohistochemical stains (i.e.﻿﻿ GLUT1, PKM2, LDHA, MCT4, p53, and PTEN), and could prove that the Warburg-high subtype is associated with poorer survival outcomes.^27^ SARIFA-positivity was associated with the Warburg-high subtype.^30^ While both SARIFA-status and Warburg-subtyping exhibited prognostic significance in CRC patients﻿, SARIFA-status demonstrated a higher prognostic value compared with Warburg-subtyping in our previous study.^30^ In line with this, compared with SARIFA-status, Warburg-subtyping was not associated with significant survival differences within mutually exclusive mutational subgroups.^28^ However, there was a significant interaction between Warburg-subtyping and adjuvant therapy for CRC-specific and overall survival,^53^ which was not the case for SARIFA-status in our current analyses. These results indicate that SARIFA-status and Warburg-subtyping could complement each other as novel “*metabolic* *”* biomarkers.

The main strengths of our presented work are the use of a large population-based series of incident CRC patients, the nearly complete and long-term follow-up, and the availability of DNA and tumor material for many CRC patients, which enabled us to form sufficiently large (molecular) subgroups. Nevertheless, our study has some limitations. First, only one﻿ representative digitized tumor slide was available for SARIFA classification. Even though we have already shown that assessing SARIFA-status on a﻿ single﻿ representative tumor slide^14,16^ is reliable, frequency of SARIFA-positive cases could potentially be a bit higher if all tumor slides were available and especially if tumors were completely embedded. Second, detailed data regarding exact therapy regimens or dosages were not available. Third, we did not adjust for multiple testing. Fourth, most CRC patients in the NLCS, with patients diagnosed in the period 1986–2006, were treated with surgery only; the frequency of adjuvant chemotherapy usage is likely to be higher in modern cohorts. However, the limited number of patients treated with adjuvant therapy was representative for this time period (1986–2006^54^) Fifth, immunotherapy or targeted therapy, which could be also of interest with regard to SARIFA-status and are﻿ nowadays applied regularly in CRC patients (especially in recurrent or metastatic disease settings^5,55^), were not part of the therapy regimens. Sixth, MMR/MSI testing relied here only on MLH1 and MSH2 immunohistochemistry, which misses out on cases that are only deficient for PMS2 or MSH6. However, it has been described that IHC analysis of MLH1 and MSH2 expression is a reliable method for the detection of the vast majority of patients with MSI CRC.^56^

## CONCLUSIONS

In conclusion, the novel H&E-based histopathological SARIFA-status maintained its prognostic value even within molecular subgroups based on *BRAF*, *RAS*, and MMR status, which proves the potential of SARIFA-status to improve stratification of CRC patients even beyond clinically already ﻿used molecular tests. Compared with molecular testing, SARIFA-status can be assessed easily and reliably on routine histological slides without additional costs (except minimal pathologist’s time) or delay in turnaround time. Moreover, the presence of SARIFA may be a novel potential histopathological tool to predict response from adjuvant therapy as patients with SARIFA-positive CRC showed a CRC-specific survival benefit from adjuvant therapy, which was not observed for patients with﻿ SARIFA-negative CRCs. Even though the interaction between treatment and SARIFA-status was not statistically significant, this finding may be seen as a first sign of a potential differential treatment response. However, as our results are based on an exploratory analysis of observational data, these findings should be interpreted with caution and future studies are necessary to validate and build upon our findings. As SARIFA-positivity is closely linked to a distinct tumor biology with upregulation of lipid metabolism, altered immunity, and stromal changes, further studies are warranted to explore the potential of not only conventional (radio-)chemotherapy but also immunotherapy, targeted therapy, and/or even novel drugs directly targeting lipid metabolism in SARIFA-positive CRCs.

## Supplementary Material
