# Supplementary material for: Prognostic and Predictive Value of SARIFA-status Within Molecular Subgroups of Colorectal Cancer: Insights From the Netherlands Cohort Study
Source: Am J Surg Pathol. 2025 May 9;49(9):956–69. doi: 10.1097/PAS.0000000000002408 (PMC12352556; doi:10.1097/PAS.0000000000002408)
Supplement: Supplementary file 1 [file pas-49-956-s001.docx]

**
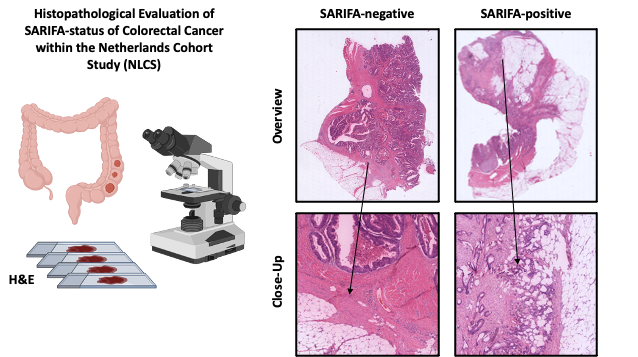
**

**Supplementary Figure S1** - Histopathological Evaluation of SARIFA-status. SARIFA-status was established on digitised H&E stained WSI of CRC resection specimens. SARIFA-positivity is defined as direct tumour-adipocyte interaction at the invasion front. If one tumour gland or at least five tumour cells show a direct contact with adipocytes, the whole case is classified as SARIFA-positive. In SARIFA-negative CRCs tumour cells and adipocytes are separated by (desmoplastic) stroma and/or an inflammatory infiltrate. All digitised slides were accessed using *QuPath* (<https://qupath.github.io/>). SARIFA-status can be assessed on routinely available histopathologic H&E slides. For all cases, one representative tumour slide was available. If the tumour-adipocyte interface was not depicted, the case was excluded (SARIFA-status cannot be established reliably when only superficial tumour parts are displayed). *Created with BioRender.com. CRC,* colorectal cancer*; SARIFA,* Stroma AReactive Invasion Front Areas*; H&E,* haematoxylin and eosin*; NLCS,* Netherlands Cohort Study*; WSI,* whole slide images.

**Supplementary Figure S2** - Examples of 2x SARIFA-positive and 2x SARIFA-negative CRC cases within the NLCS. *Left Panel:* SARIFA-negative cases with no direct tumour-adipocyte interaction at the invasion front. Tumour cells and adipocytes are separated by (desmoplastic) stroma and/or an inflammatory infiltrate. *Right Panel:* SARIFA-positive cases with direct tumour-adipocyte interaction at the invasion front. Interestingly, SARIFA-positivity is not restricted to a certain histomorphologic subtype as here also a mucinous CRC with signet-ring cells shows SARIFA-positivity. SARIFA-status can be easily assessed on routine H&E-stained tissue slides, as shown here.
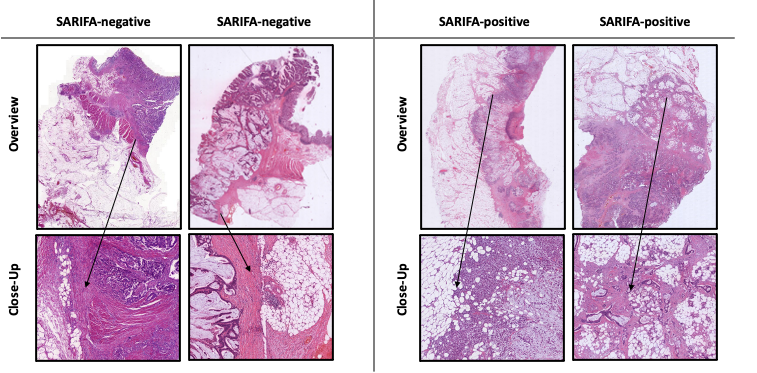
 *CRC,* colorectal cancer*; H&E,* haematoxylin and eosin*; NLCS,* Netherlands Cohort Study*, SARIFA,* Stroma AReactive Invasion Front Areas.

**
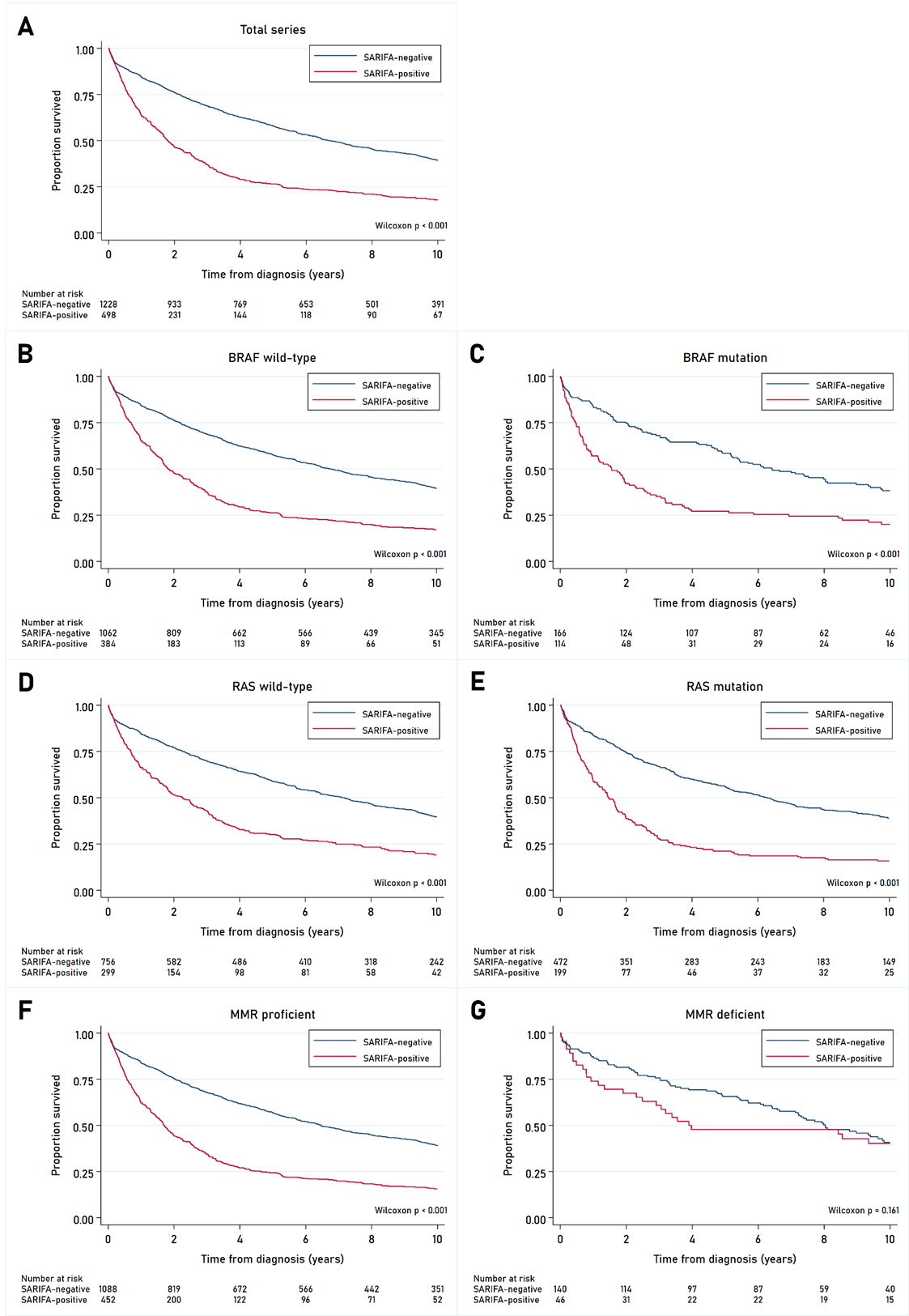
**

**Supplementary Figure S3** – Univariable Kaplan-Meier curves showing the overall survival of colorectal cancer patients within the Netherlands Cohort Study (NLCS; 1986-2006) according to SARIFA-status or **(A)** the total series of CRC patients, as well as *within* prognostically relevant molecular subgroups: **(B)** *BRAF*_wt_, **(C)** *BRAF*_mut_, **(D)** *RAS*_wt_, **(E)** *RAS*_mut_, **(F)** pMMR, **(G)** dMMR. *SARIFA*, Stroma AReactive Invasion Front Areas*; BRAF*, V-Raf Murine Sarcoma Viral Oncogene Homolog B; *RAS*, Rat sarcoma; *RT*, radiotherapy; *CHT*, chemotherapy; *MMR*, mismatch repair.


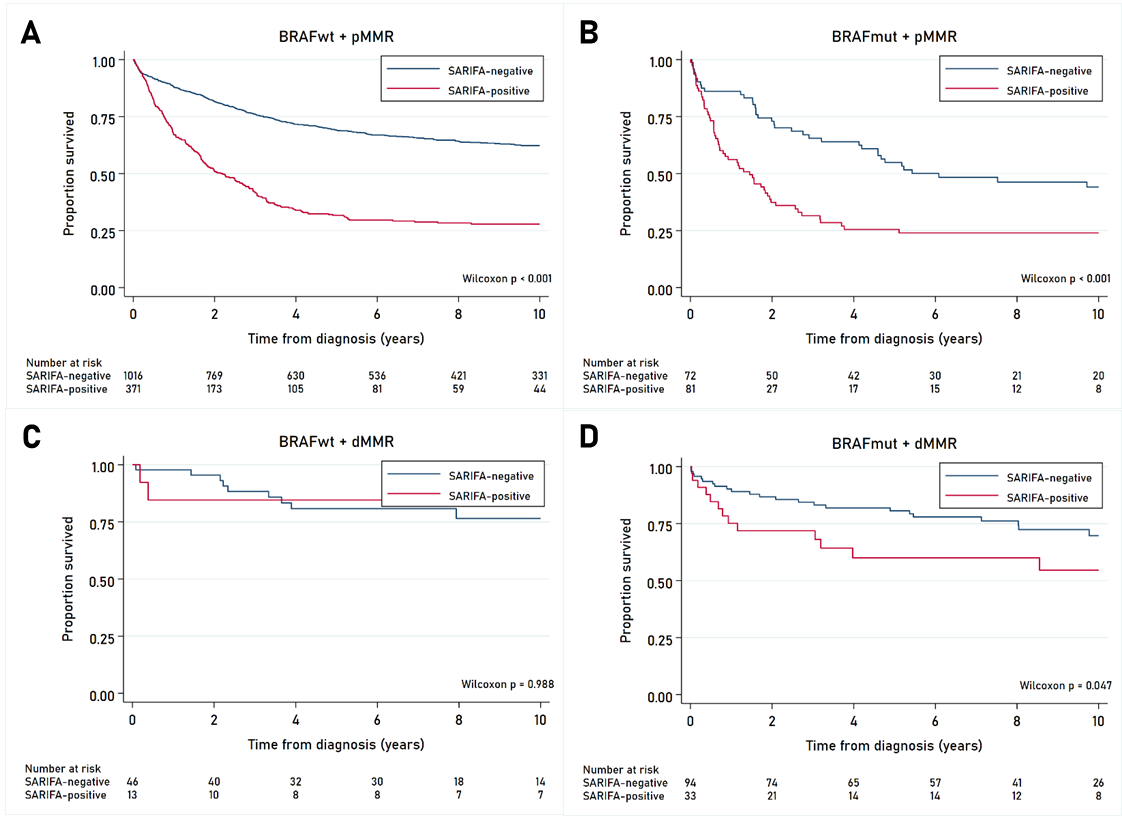


**Supplementary Figure S4 -** Univariable Kaplan-Meier curves showing the CRC-specific survival of colorectal cancer patients within the Netherlands Cohort Study (NLCS; 1986-2006; n = 1,726) according to subgroups based on both BRAF and MMR status: **(A)** BRAF_wt_ + pMMR, **(B)** BRAF_mut_ + pMMR, **(C)** BRAF_wt_ + dMMR, and **(D)** BRAF_mut_ + dMMR. *SARIFA*, Stroma AReactive Invasion Front Areas; *BRAF*, V-Raf Murine Sarcoma Viral Oncogene Homolog B; *RAS*, Rat sarcoma; *MMR*, mismatch repair.

**
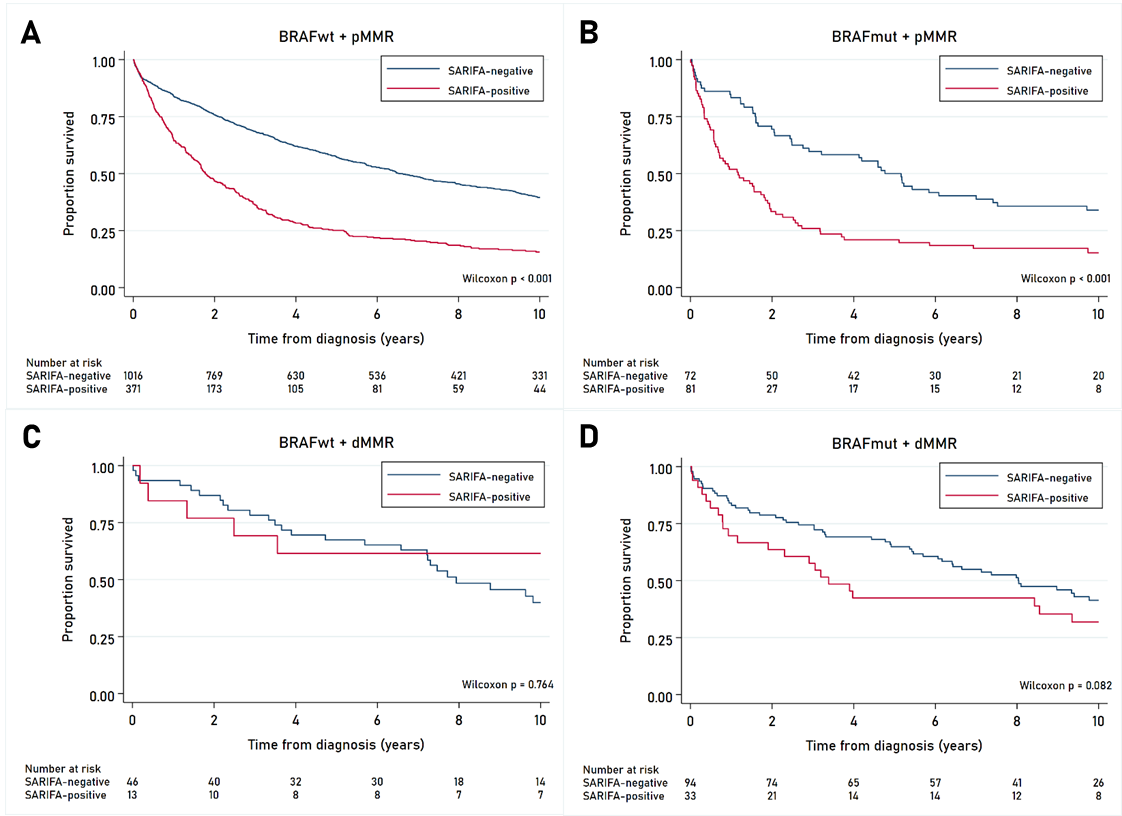
**

**Supplementary Figure S5 -** Univariable Kaplan-Meier curves showing the overall survival of colorectal cancer patients within the Netherlands Cohort Study (NLCS; 1986-2006; n = 1,726) according to subgroups based on both BRAF and MMR status: **(A)** BRAF_wt_ + pMMR, **(B)** BRAF_mut_ + pMMR, **(C)** BRAF_wt_ + dMMR, and **(D)** BRAF_mut_ + dMMR. *SARIFA*, Stroma AReactive Invasion Front Areas; *BRAF*, V-Raf Murine Sarcoma Viral Oncogene Homolog B; *RAS*, Rat sarcoma; *MMR*, mismatch repair.


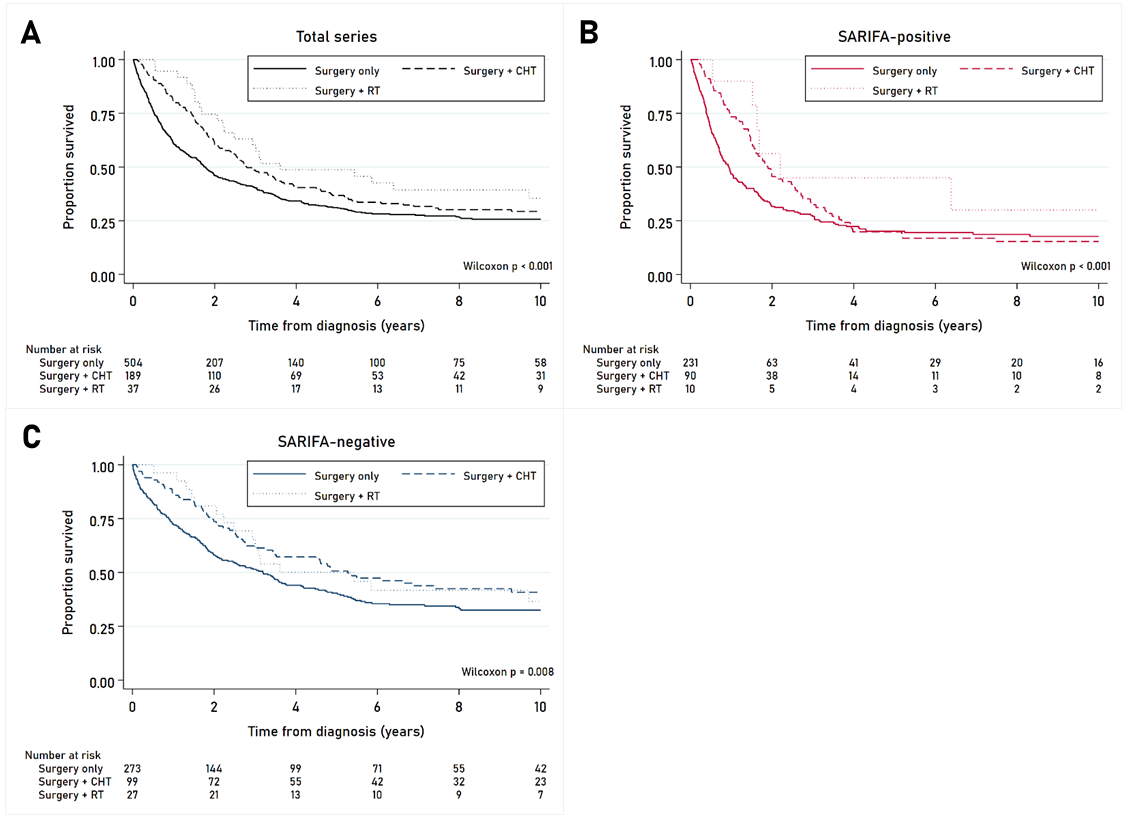


**Supplementary Figure S6** – Univariable Kaplan-Meier curves showing CRC-specific survival of colorectal cancer patients within the Netherlands Cohort Study (NLCS; 1986-2006) according to therapeutic intervention (surgery, surgery + adjuvant radiotherapy, surgery + adjuvant chemotherapy) for **(A)** the total series of colorectal cancer patients, **(B)** SARIFA-negative patients, **(C)** SARIFA-positive patients. *SARIFA*, Stroma AReactive Invasion Front Areas; *CHT*, chemotherapy; *RT*, radiotherapy.


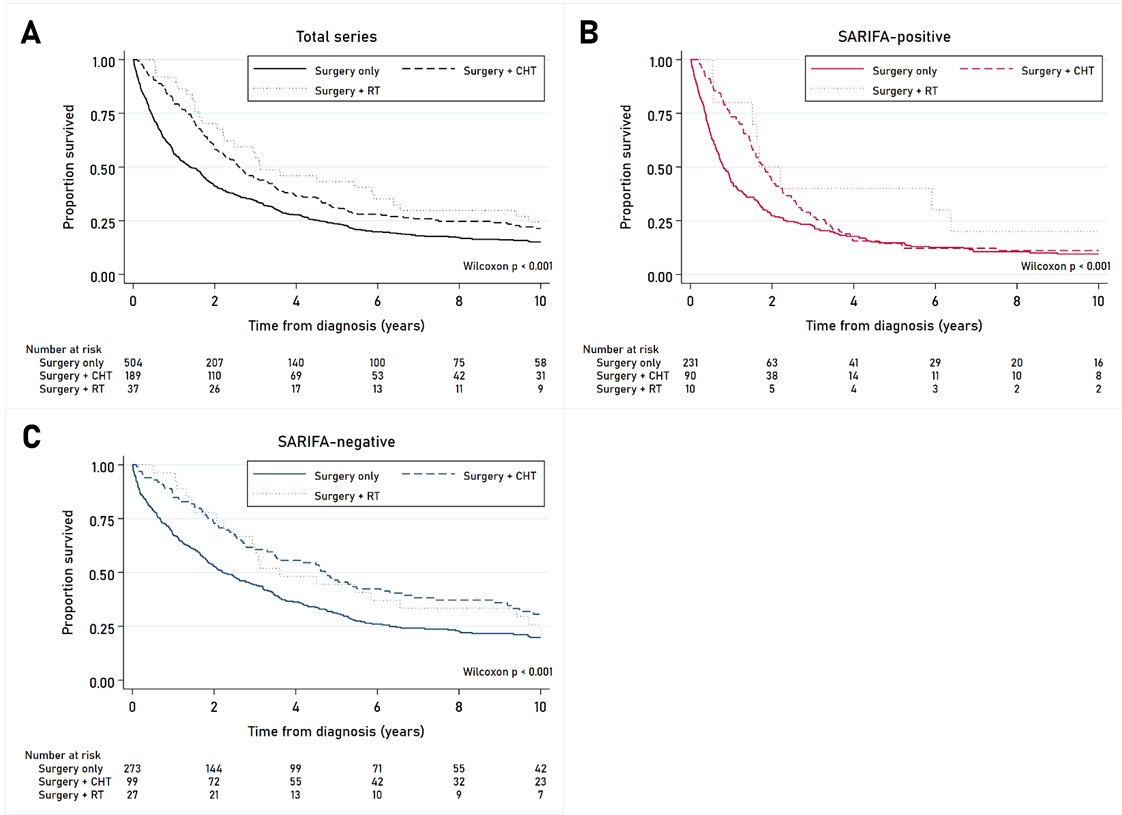


**Supplementary Figure S7** – Univariable Kaplan-Meier curves showing overall survival of colorectal cancer patients within the Netherlands Cohort Study (NLCS; 1986-2006) according to therapeutic intervention (surgery, surgery + adjuvant radiotherapy, surgery + adjuvant chemotherapy) for **(A)** the total series of colorectal cancer patients, **(B)** SARIFA-negative patients, **(C)** SARIFA-positive patients. *SARIFA*, Stroma AReactive Invasion Front Areas; *CHT*, chemotherapy; *RT*, radiotherapy.
