# Supplementary material for: Prognostic and Predictive Value of SARIFA-status Within Molecular Subgroups of Colorectal Cancer: Insights From the Netherlands Cohort Study
Source: Am J Surg Pathol. 2025 May 9;49(9):956–69. doi: 10.1097/PAS.0000000000002408 (PMC12352556; doi:10.1097/PAS.0000000000002408)
Supplement: Supplementary file 2 [file pas-49-956-s002.docx]

**Supplementary Table S1 -** Clinical characteristics of the total series of colorectal cancer patients within the Netherlands Cohort Study (NLCS; 1986-2006), as well as according to SARIFA-status (SARIFA-positive and SARIFA-negative; *n*  = 1,726).

|  |  | **Total series of CRC patients**  **(*n* = 1,726)** |  | **SARIFA-status** | | |
| --- | --- | --- | --- | --- | --- | --- |
|  |  |  |  | **SARIFA-negative**  **(*n* = 1228)** | **SARIFA-positive**  **(*n* = 498)** | ***p*-value^a^** |
|  |  |  |  |  |  |  |
| **Age at diagnosis in years, median (range)** | | 74.0 (55.0-89.0) |  | 74.0 (55.0-89.0) | 74.0 (55.0-88.0) | 0.292^b^ |
| **Sex, *n* (%)** | |  |  |  |  |  |
|  | Men | 961 (55.7) |  | 692 (56.4) | 269 (54.0) |  |
|  | Women | 765 (44.3) |  | 536 (43.7) | 229 (46.0) | 0.376 |
| **Tumour location, *n* (%)** | |  |  |  |  |  |
|  | Colon | 1274 (73.8) |  | 854 (69.5) | 420 (84.3) |  |
|  | Rectosigmoid | 181 (10.5) |  | 145 (11.8) | 36 (7.2) |  |
|  | Rectum | 271 (15.7) |  | 229 (18.7) | 42 (8.4) | <0.001 |
| **pTNM stage, *n* (%)** | |  |  |  |  |  |
|  | I | 312 (18.1) |  | 306 (24.9) | 6 (1.2) |  |
|  | II | 661 (38.3) |  | 509 (41.5) | 152 (30.5) |  |
|  | III | 458 (26.5) |  | 278 (22.6) | 180 (36.1) |  |
|  | IV | 247 (14.3) |  | 103 (8.4) | 144 (28.9) |  |
|  | Unknown | 48 (2.8) |  | 32 (2.6) | 16 (3.2) | <0.001 |
| **Tumour extension (pT), *n* (%)** | |  |  |  |  |  |
|  | T1 | 68 (3.9) |  | 67 (5.5) | 1 (0.2) |  |
|  | T2 | 301 (17.4) |  | 292 (23.8) | 9 (1.8) |  |
|  | T3 | 1141 (66.1) |  | 763 (62.1) | 378 (75.9) |  |
|  | T4 | 166 (9.6) |  | 74 (6.0) | 92 (18.5) |  |
|  | Unknown | 50 (2.9) |  | 32 (2.6) | 18 (3.6) | <0.001 |
| **Lymph node involvement (pN), *n* (%)** | |  |  |  |  |  |
|  | N0 | 892 (51.7) |  | 728 (59.3) | 164 (32.9) |  |
|  | N+ | 634 (36.7) |  | 344 (28.0) | 290 (58.2) |  |
|  | Unknown | 200 (11.6) |  | 156 (12.7) | 44 (8.8) | <0.001 |
| **Differentiation grade, *n* (%)** | |  |  |  |  |  |
|  | Well | 147 (8.5) |  | 120 (9.8) | 27 (5.4) |  |
|  | Moderate | 1131 (65.5) |  | 841 (68.5) | 290 (58.2) |  |
|  | Poor/undifferentiated | 315 (18.3) |  | 165 (13.4) | 150 (30.1) |  |
|  | Unknown | 133 (7.7) |  | 102 (8.3) | 31 (6.2) | <0.001 |
| **Adjuvant therapy, *n* (%)** | |  |  |  |  |  |
|  | No | 1420 (82.3) |  | 1042 (84.9) | 378 (75.9) |  |
|  | Yes | 288 (16.7) |  | 175 (14.3) | 113 (22.7) |  |
|  | Unknown | 18 (1.0) |  | 11 (0.9) | 7 (1.4) | <0.001 |
| *CRC,* colorectal cancer; *SARIFA*, Stroma AReactive Invasion Front Areas*; pTNM,* pathological tumour-node-metastasis stage  ^a^*P*-value for the χ2 test, unless otherwise specified  ^b^*P*-value for the Kruskall-Wallis test | | | | | | |
