# Supplementary material for: Prognostic and Predictive Value of SARIFA-status Within Molecular Subgroups of Colorectal Cancer: Insights From the Netherlands Cohort Study
Source: Am J Surg Pathol. 2025 May 9;49(9):956–69. doi: 10.1097/PAS.0000000000002408 (PMC12352556; doi:10.1097/PAS.0000000000002408)
Supplement: Supplementary file 3 [file pas-49-956-s003.docx]

**Supplementary Table S2 -** Univariable and multivariable-adjusted hazard ratios for associations between SARIFA-status and survival of colorectal cancer cases within the Netherlands Cohort Study (NLCS, 1986-2006) *within* subgroups based on mismatch repair status and *BRAF* mutational status (*n* = 2,236).

|  |  | **N** |  | **CRC-specific survival** | | |  | **Overall survival** | | |
| --- | --- | --- | --- | --- | --- | --- | --- | --- | --- | --- |
|  |  |  |  | **CRC-deaths (%)** | **HR (95% CI)** | |  | **Deaths (%)** | **HR (95% CI)** | |
|  |  |  |  |  | **Univariable** | **Multivariable-adjusted^a^** |  |  | **Univariable** | **Multivariable-adjusted^a^** |
| **Overall** | |  |  |  |  |  |  |  |  |  |
|  | SARIFA-negative | 1228 |  | 411 (33.5) | 1.00 (ref) | 1.00 (ref) |  | 729 (59.4) | 1.00 (ref) | 1.00 (ref) |
|  | SARIFA-positive | 498 |  | 319 (64.1) | 2.76 (2.38-3.20) | 1.59 (1.35-1.87) |  | 405 (81.3) | 2.11 (1.87-2.39) | 1.42 (1.24-1.63) |
|  | SARIFA-unknown | 510 |  | 197 (38.6) | 1.28 (1.08-1.52) | 1.21 (1.01-1.45) |  | 324 (63.5) | 1.20 (1.05-1.36) | 1.15 (1.00-1.32) |
| ***BRAF*_wt_ + pMMR** | |  |  |  |  |  |  |  |  |  |
|  | SARIFA-negative | 1016 |  | 342 (33.7) | 1.00 (ref) | 1.00 (ref) |  | 604 (59.4) | 1.00 (ref) | 1.00 (ref) |
|  | SARIFA-positive | 371 |  | 247 (66.6) | 2.88 (2.44-3.40) | 1.67 (1.40-1.98) |  | 310 (83.6) | 2.21 (1.92-2.54) | 1.47 (1.27-1.70) |
|  | SARIFA-unknown | 408 |  | 157 (38.5) | 1.25 (1.03-1.51) | 1.15 (0.95-1.39) |  | 253 (62.0) | 1.14 (0.99-1.33) | 1.09 (0.94-1.27) |
| ***BRAF*_mut_ + pMMR** | |  |  |  |  |  |  |  |  |  |
|  | SARIFA-negative | 72 |  | 37 (51.4) | 1.00 (ref) | 1.00 (ref) |  | 47 (65.3) | 1.00 (ref) | 1.00 (ref) |
|  | SARIFA-positive | 81 |  | 57 (70.4) | 2.11 (1.39-3.20) | 1.83 (1.15-2.91) |  | 68 (84.0) | 2.06 (1.42-3.00) | 2.03 (1.34-3.08) |
|  | SARIFA-unknown | 36 |  | 20 (55.6) | 1.36 (0.79-2.34) | 1.87 (1.04-3.36) |  | 28 (77.8) | 1.48 (0.93-2.37) | 1.96 (1.18-3.24) |
| **BRAF_wt_ + dMMR** | |  |  |  |  |  |  |  |  |  |
|  | SARIFA-negative | 46 |  | 9 (19.6) | 1.00 (ref) | 1.00 (ref) |  | 26 (56.5) | 1.00 (ref) | 1.00 (ref) |
|  | SARIFA-positive | 13 |  | 2 (15.4) | 0.84 (0.18-3.89) | 0.70 (0.12-3.96) |  | 5 (38.5) | 0.69 (0.26-1.80) | 1.00 (0.35-2.88) |
|  | SARIFA-unknown | 24 |  | 5 (20.8) | 1.13 (0.38-3.38) | 1.17 (0.35-3.90) |  | 15 (62.5) | 1.23 (0.65-2.32) | 1.32 (0.65-2.67) |
| **BRAF_mut_ + dMMR** | |  |  |  |  |  |  |  |  |  |
|  | SARIFA-negative | 94 |  | 23 (24.5) | 1.00 (ref) | 1.00 (ref) |  | 52 (55.3) | 1.00 (ref) | 1.00 (ref) |
|  | SARIFA-positive | 33 |  | 13 (39.4) | 1.87 (0.95-3.69) | 1.51 (0.72-3.16) |  | 22 (66.7) | 1.45 (0.88-2.39) | 1.32 (0.76-2.29) |
|  | SARIFA-unknown | 42 |  | 15 (35.7) | 1.75 (0.91-3.35) | 1.39 (0.68-2.82) |  | 28 (66.7) | 1.50 (0.95-2.38) | 1.44 (0.88-2.34) |
| *CRC*, colorectal cancer; *HR*, hazard ratio; *CI*, confidence interval; *SARIFA*, Stroma AReactive Invasion Front Areas; *BRAF*, V-Raf Murine Sarcoma Viral Oncogene Homolog B; *wt*, wild-type; *mut*, mutation; *dMMR*, mismatch repair deficient; *pMMR*, mismatch repair proficient  ^a^Adjusted for age at diagnosis (years), sex (male, female), tumour location (colon, rectosigmoid, rectum), pTNM stage (III, IV, unknown), differentiation grade (well, moderate, poor/undifferentiated, unknown), and adjuvant therapy (no, yes, unknown) | | | | | | | | | | |
