# Supplementary material for: Prognostic and Predictive Value of SARIFA-status Within Molecular Subgroups of Colorectal Cancer: Insights From the Netherlands Cohort Study
Source: Am J Surg Pathol. 2025 May 9;49(9):956–69. doi: 10.1097/PAS.0000000000002408 (PMC12352556; doi:10.1097/PAS.0000000000002408)
Supplement: Supplementary file 4 [file pas-49-956-s004.docx]

**Supplementary Table S3 -** Univariable and multivariable-adjusted hazard ratios for associations between SARIFA-status and survival of pT3 (n = 1,455) and pT4 (n = 220) colorectal cancer patients within the Netherlands Cohort Study (NLCS, 1986-2006).

|  | | | N |  | **CRC-specific survival** | | |  | **Overall survival** | | |
| --- | --- | --- | --- | --- | --- | --- | --- | --- | --- | --- | --- |
|  | | |  |  | **CRC deaths (%)** | **HR (95% CI)** | |  | **Deaths (%)** | **HR (95% CI)** | |
|  | | |  |  |  | **Univariable** | **Multivariable-adjusted^a^** |  |  | **Univariable** | **Multivariable-adjusted^a^** |
|  | | |  |  |  |  |  |  |  |  |  |
| **pT3** | | |  |  |  |  |  |  |  |  |  |
|  |  | SARIFA-negative | 763 |  | 276 (36.2) | 1.00 (ref) | 1.00 (ref) |  | 469 (61.5) | 1.00 (ref) | 1.00 (ref) |
|  |  | SARIFA-positive | 378 |  | 224 (59.3) | 2.09 (1.75-2.50) | 1.49 (1.24-1.78) |  | 295 (78.0) | 1.71 (1.48-1.98) | 1.36 (1.17-1.58) |
|  |  | SARIFA-unknown | 314 |  | 129 (41.1) | 1.26 (1.02-1.55) | 1.16 (0.94-1.44) |  | 213 (67.8) | 1.24 (1.05-1.46) | 1.17 (0.99-1.38) |
| **pT4** | | |  |  |  |  |  |  |  |  |  |
|  |  | SARIFA-negative | 74 |  | 44 (59.5) | 1.00 (ref) | 1.00 (ref) |  | 60 (81.1) | 1.00 (ref) | 1.00 (ref) |
|  |  | SARIFA-positive | 92 |  | 71 (77.2) | 2.18 (1.49-3.20) | 1.79 (1.19-2.69) |  | 84 (91.3) | 1.97 (1.41-2.77) | 1.74 (1.21-2.50) |
|  |  | SARIFA-unknown | 54 |  | 33 (61.1) | 1.32 (0.84-2.08) | 1.20 (0.75-1.91) |  | 44 (81.5) | 1.29 (0.87-1.90) | 1.25 (0.84-1.87) |

*CRC*, colorectal cancer; *HR*, hazard ratio; *CI*, confidence interval; *SARIFA*, Stroma AReactive Invasion Front Areas.

^a^Adjusted for age at diagnosis (years), sex (male, female), tumour location (colon, rectosigmoid, rectum), pTNM stage (I, II, III, IV, unknown), differentiation grade (well, moderate, poor/undifferentiated, unknown), adjuvant therapy (no, yes, unknown), and MMR status (proficient, deficient).
