# Supplementary material for: Prognostic and Predictive Value of SARIFA-status Within Molecular Subgroups of Colorectal Cancer: Insights From the Netherlands Cohort Study
Source: Am J Surg Pathol. 2025 May 9;49(9):956–69. doi: 10.1097/PAS.0000000000002408 (PMC12352556; doi:10.1097/PAS.0000000000002408)
Supplement: Supplementary file 5 [file pas-49-956-s005.docx]

**Supplementary Table S4 –** Univariable and multivariable-adjusted hazard ratios for associations between SARIFA-status and survival of pT3 and pT4 colorectal cancer cases within the Netherlands Cohort Study (NLCS, 1986-2006) *within* molecular subgroups (*BRAF*_wt_/*BRAF*_mut_, *RAS*_wt_/*RAS*_mut_, pMMR/dMMR; *n* = 1,675).

|  | | | N |  | **CRC-specific survival** | | |  | **Overall survival** | | |
| --- | --- | --- | --- | --- | --- | --- | --- | --- | --- | --- | --- |
|  | | |  |  | **CRC deaths (%)** | **HR (95% CI)** | |  | **Deaths (%)** | **HR (95% CI)** | |
|  | | |  |  |  | **Univariable** | **Multivariable-adjusted^a^** |  |  | **Univariable** | **Multivariable-adjusted^a^** |
| **Overall** | | |  |  |  |  |  |  |  |  |  |
|  |  | SARIFA-negative | 837 |  | 320 (38.2) | 1.00 (ref) | 1.00 (ref) |  | 529 (63.2) | 1.00 (ref) | 1.00 (ref) |
|  |  | SARIFA-positive | 470 |  | 295 (62.8) | 2.23 (1.90-2.61) | 1.60 (1.35-1.90) |  | 379 (80.6) | 1.82 (1.60-2.08) | 1.44 (1.25-1.67) |
|  |  | SARIFA-unknown | 368 |  | 162 (44.0) | 1.31 (1.09-1.59) | 1.26 (1.03-1.54) |  | 257 (69.8) | 1.27 (1.10 – 1.48) | 1.24 (1.06-1.45) |
|  |  |  |  |  |  |  |  |  |  |  |  |
| ***BRAF*** | | |  |  |  |  |  |  |  |  |  |
|  | **Wild-type** | |  |  |  |  |  |  |  |  |  |
|  |  | SARIFA-negative | 705 |  | 271 (38.4) | 1.00 (ref) | 1.00 (ref) |  | 453 (64.3) | 1.00 (ref) | 1.00 (ref) |
|  |  | SARIFA-positive | 362 |  | 230 (63.5) | 2.22 (1.86-2.65) | 1.56 (1.29-1.88) |  | 294 (81.2) | 1.79 (1.54-2.07) | 1.36 (1.16-1.60) |
|  |  | SARIFA-unknown | 303 |  | 129 (42.6) | 1.22 (0.99-1.51) | 1.19 (0.95-1.48) |  | 204 (67.3) | 1.16 (0.98-1.37) | 1.14 (0.96-1.36) |
|  | **Mutation** | |  |  |  |  |  |  |  |  |  |
|  |  | SARIFA-negative | 132 |  | 49 (37.1) | 1.00 (ref) | 1.00 (ref) |  | 76 (57.6) | 1.00 (ref) | 1.00 (ref) |
|  |  | SARIFA-positive | 108 |  | 65 (60.2) | 2.27 (1.56-3.30) | 1.77 (1.17-2.70) |  | 85 (78.7) | 2.02 (1.48-2.76) | 1.81 (1.28-2.56) |
|  |  | SARIFA-unknown | 65 |  | 33 (50.8) | 1.80 (1.16-2.81) | 1.98 (1.22-3.21) |  | 53 (81.5) | 1.95 (1.37-2.77) | 2.10 (1.43-3.08) |
|  |  |  |  |  |  |  |  |  |  |  |  |
| ***RAS*** | | |  |  |  |  |  |  |  |  |  |
|  | **Wild-type** | |  |  |  |  |  |  |  |  |  |
|  |  | SARIFA-negative | 513 |  | 188 (36.6) | 1.00 (ref) | 1.00 (ref) |  | 321 (62.6) | 1.00 (ref) | 1.00 (ref) |
|  |  | SARIFA-positive | 281 |  | 161 (57.3) | 2.04 (1.65-2.52) | 1.46 (1.16-1.84) |  | 222 (79.0) | 1.74 (1.46-2.06) | 1.47 (1.22-1.77) |
|  |  | SARIFA-unknown | 229 |  | 97 (42.4) | 1.30 (1.01-1.66) | 1.06 (0.81-1.38) |  | 159 (69.4) | 1.25 (1.04-1.52) | 1.13 (0.92-1.38) |
|  | **Mutation** | |  |  |  |  |  |  |  |  |  |
|  |  | SARIFA-negative | 324 |  | 132 (40.7) | 1.00 (ref) | 1.00 (ref) |  | 208 (64.2) | 1.00 (ref) | 1.00 (ref) |
|  |  | SARIFA-positive | 189 |  | 134 (70.9) | 2.53 (1.98-3.22) | 1.89 (1.45-2.46) |  | 157 (83.1) | 1.98 (1.60-2.43) | 1.47 (1.17-1.84) |
|  |  | SARIFA-unknown | 139 |  | 65 (46.8) | 1.35 (1.01-1.82) | 1.69 (1.24-2.31) |  | 98 (70.5) | 1.30 (1.03-1.66) | 1.53 (1.19-1.96) |
|  |  |  |  |  |  |  |  |  |  |  |  |
| **Mismatch repair (MMR) status** | | |  |  |  |  |  |  |  |  |  |
|  | **Proficient (pMMR)** | |  |  |  |  |  |  |  |  |  |
|  |  | SARIFA-negative | 726 |  | 291 (40.1) | 1.00 (ref) | 1.00 (ref) |  | 467 (64.3) | 1.00 (ref) | 1.00 (ref) |
|  |  | SARIFA-positive | 426 |  | 280 (65.7) | 2.27 (1.92-2.67) | 1.61 (1.35-1.93) |  | 352 (82.6) | 1.88 (1.63-2.16) | 1.44 (1.24-1.68) |
|  |  | SARIFA-unknown | 311 |  | 143 (46.0) | 1.30 (1.06-1.59) | 1.25 (1.01-1.54) |  | 216 (69.5) | 1.23 (1.04-1.44) | 1.21 (1.02-1.43) |
|  | **Deficient (dMMR)** | |  |  |  |  |  |  |  |  |  |
|  |  | SARIFA-negative | 111 |  | 29 (26.1) | 1.00 (ref) | 1.00 (ref) |  | 62 (55.9) | 1.00 (ref) | 1.00 (ref) |
|  |  | SARIFA-positive | 44 |  | 15 (34.1) | 1.49 (0.80-2.78) | 1.37 (0.68-2.74) |  | 27 (61.4) | 1.26 (0.80-1.97) | 1.36 (0.82-2.25) |
|  |  | SARIFA-unknown | 57 |  | 19 (33.3) | 1.51 (0.85-2.69) | 1.52 (0.79-2.92) |  | 41 (71.9) | 1.60 (1.08-2.38) | 1.53 (0.99-2.37) |
| *CRC*, colorectal cancer; *HR*, hazard ratio; *CI*, confidence interval; *SARIFA*, Stroma AReactive Invasion Front Areas; *BRAF*, V-Raf Murine Sarcoma Viral Oncogene Homolog B; *RAS*, Rat sarcoma  ^a^Adjusted for age at diagnosis (years), sex (male, female), tumour location (colon, rectosigmoid, rectum), pTNM stage (I, II, III, IV, unknown), differentiation grade (well, moderate, poor/undifferentiated, unknown), adjuvant therapy (no, yes, unknown), and MMR status (proficient, deficient). | | | | | | | | | | | |
