# Supplementary material for: Prognostic and Predictive Value of SARIFA-status Within Molecular Subgroups of Colorectal Cancer: Insights From the Netherlands Cohort Study
Source: Am J Surg Pathol. 2025 May 9;49(9):956–69. doi: 10.1097/PAS.0000000000002408 (PMC12352556; doi:10.1097/PAS.0000000000002408)
Supplement: Supplementary file 6 [file pas-49-956-s006.docx]

**Supplementary Table S5 -** Univariable and multivariable-adjusted hazard ratios for associations between SARIFA-status and survival of pT3-4 colorectal cancer patients within the Netherlands Cohort Study (NLCS, 1986-2006) *within* subgroups based on mismatch repair status and *BRAF* mutational status (*n* = 1,675).

|  |  | **N** |  | **CRC-specific survival** | | |  | **Overall survival** | | |
| --- | --- | --- | --- | --- | --- | --- | --- | --- | --- | --- |
|  |  |  |  | **CRC-deaths (%)** | **HR (95% CI)** | |  | **Deaths (%)** | **HR (95% CI)** | |
|  |  |  |  |  | **Univariable** | **Multivariable-adjusted^a^** |  |  | **Univariable** | **Multivariable-adjusted^a^** |
| ***BRAF*_wt_ + pMMR** | |  |  |  |  |  |  |  |  |  |
|  | SARIFA-negative | 671 |  | 264 (39.3) | 1.00 (ref) | 1.00 (ref) |  | 434 (64.7) | 1.00 (ref) | 1.00 (ref) |
|  | SARIFA-positive | 350 |  | 228 (65.1) | 2.24 (1.88-2.68) | 1.56 (1.29-1.89) |  | 289 (82.6) | 1.83 (1.57-2.12) | 1.38 (1.18-1.61) |
|  | SARIFA-unknown | 280 |  | 124 (44.3) | 1.25 (1.01-1.55) | 1.18 (0.94-1.48) |  | 189 (67.5) | 1.16 (0.98-1.38) | 1.08 (0.91-1.29) |
| ***BRAF*_mut_ + pMMR** | |  |  |  |  |  |  |  |  |  |
|  | SARIFA-negative | 55 |  | 27 (49.1) | 1.00 (ref) | 1.00 (ref) |  | 33 (60.0) | 1.00 (ref) | 1.00 (ref) |
|  | SARIFA-positive | 76 |  | 52 (68.4) | 2.07 (1.30-3.31) | 2.11 (1.24-3.57) |  | 63 (82.9) | 2.15 (1.41-3.29) | 2.24 (1.41-3.57) |
|  | SARIFA-unknown | 31 |  | 19 (61.3) | 1.67 (0.93-3.01) | 2.46 (1.29-4.71) |  | 27 (87.1) | 1.99 (1.20-3.31) | 2.79 (1.61-4.83) |
| **BRAF_wt_ + dMMR** | |  |  |  |  |  |  |  |  |  |
|  | SARIFA-negative | 34 |  | 7 (20.6) | 1.00 (ref) | 1.00 (ref) |  | 19 (55.9) | 1.00 (ref) | 1.00 (ref) |
|  | SARIFA-positive | 12 |  | 2 (16.7) | 0.89 (0.18-4.27) | 0.90 (0.13-6.30) |  | 5 (41.7) | 0.75 (0.28-2.01) | 1.04 (0.35-3.11) |
|  | SARIFA-unknown | 23 |  | 5 (21.7) | 1.10 (0.35-3.46) | 2.29 (0.52-10.13) |  | 15 (65.2) | 1.28 (0.65-2.52) | 1.38 (0.66-2.88) |
| **BRAF_mut_ + dMMR** | |  |  |  |  |  |  |  |  |  |
|  | SARIFA-negative | 77 |  | 22 (28.6) | 1.00 (ref) | 1.00 (ref) |  | 43 (55.8) | 1.00 (ref) | 1.00 (ref) |
|  | SARIFA-positive | 32 |  | 13 (40.6) | 1.67 (0.84-3.32) | 1.42 (0.67-3.02) |  | 22 (68.8) | 1.48 (0.89-2.48) | 1.35 (0.77-2.38) |
|  | SARIFA-unknown | 34 |  | 14 (41.2) | 1.87 (0.95-3.67) | 1.53 (0.72-3.27) |  | 26 (76.5) | 1.89 (1.16-3.07) | 1.71 (1.03-2.85) |
| *CRC*, colorectal cancer; *HR*, hazard ratio; *CI*, confidence interval; *SARIFA*, Stroma AReactive Invasion Front Areas; *BRAF*, V-Raf Murine Sarcoma Viral Oncogene Homolog B; *wt*, wild-type; *mut*, mutation; *dMMR*, mismatch repair deficient; *pMMR*, mismatch repair proficient  ^a^Adjusted for age at diagnosis (years), sex (male, female), tumour location (colon, rectosigmoid, rectum), pTNM stage (I, II III, IV, unknown), differentiation grade (well, moderate, poor/undifferentiated, unknown), and adjuvant therapy (no, yes, unknown) | | | | | | | | | | |
