# Supplementary material for: Prognostic and Predictive Value of SARIFA-status Within Molecular Subgroups of Colorectal Cancer: Insights From the Netherlands Cohort Study
Source: Am J Surg Pathol. 2025 May 9;49(9):956–69. doi: 10.1097/PAS.0000000000002408 (PMC12352556; doi:10.1097/PAS.0000000000002408)
Supplement: Supplementary file 7 [file pas-49-956-s007.docx]

**Supplementary Table S6** – Association between adjuvant therapy and CRC-specific and overall survival of pTNM stage II-IV colorectal cancer cases within the Netherlands Cohort Study (NLCS, 1986-2006), according to SARIFA status (SARIFA-positive and SARIFA-negative; *n* = 1,385).

|  | | **N** |  | **CRC-specific survival** | | |  | **Overall survival** | | |
| --- | --- | --- | --- | --- | --- | --- | --- | --- | --- | --- |
|  |  |  |  | **CRC deaths (%)** | **HR (95% CI)** | |  | **Deaths (%)** | **HR (95% CI)** | |
|  | |  |  |  | **Univariable** | **Multivariable-adjusted^a^** |  |  | **Univariable** | **Multivariable-adjusted^a^** |
| **Colorectal cancer** | |  |  |  |  |  |  |  |  |  |
|  | Surgery only | 1114 |  | 497 (44.6) | 1.00 (ref) | 1.00 (ref) |  | 759 (68.1) | 1.00 (ref) | 1.00 (ref) |
|  | Surgery + adjuvant therapy | 271 |  | 165 (60.9) | 1.34 (1.13-1.60) | 0.77 (0.64-0.93) |  | 202 (74.5) | 1.11 (0.95-1.29) | 0.75 (0.63-0.88) |
|  | *Surgery + adjuvant CHT* | *204* |  | *129 (63.2)* | *1.44 (1.19-1.75)* | *0.71 (0.58-0.87)* |  | *153 (75.0)* | *1.16 (0.97-1.38)* | *0.67 (0.56-0.81)* |
|  | *Surgery + adjuvant RT* | *67* |  | *36 (53.7)* | *1.08 (0.77-1.51)* | *1.12 (0.76-1.64)* |  | *49 (73.1)* | *0.97 (0.73-1.30)* | *1.15 (0.83-1.59)* |
|  |  |  |  |  |  |  |  |  |  |  |
| **SARIFA-positive** | |  |  |  |  |  |  |  |  |  |
|  | Surgery only | 372 |  | 229 (61.6) | 1.00 (ref) | 1.00 (ref) |  | 297 (79.8) | 1.00 (ref) | 1.00 (ref) |
|  | Surgery + adjuvant therapy | 109 |  | 79 (72.5) | 1.10 (0.85-1.42) | 0.61 (0.46-0.80) |  | 94 (86.2) | 1.04 (0.82-1.31) | 0.63 (0.49-0.81) |
|  | *Surgery + adjuvant CHT* | *94* |  | *72 (76.6)* | *1.20 (0.92-1.57)* | *0.60 (0.45-0.80)* |  | *83 (88.3)* | *1.12 (0.88-1.43)* | *0.61 (0.47-0.79)* |
|  | *Surgery + adjuvant RT* | *15* |  | *7 (46.7)* | *0.57 (0.27-1.21)* | *0.68 (0.30-1.54)* |  | *11 (73.3)* | *0.68 (0.37-1.25)* | *0.86 (0.33-1.67)* |
|  |  |  |  |  |  |  |  |  |  |  |
| **SARIFA-negative** | |  |  |  |  |  |  |  |  |  |
|  | Surgery only | 742 |  | 268 (36.1) | 1.00 (ref) | 1.00 (ref) |  | 462 (62.3) | 1.00 (ref) | 1.00 (ref) |
|  | Surgery + adjuvant therapy | 162 |  | 86 (53.1) | 1.44 (1.13-1.84) | 0.91 (0.70-1.19) |  | 108 (66.7) | 1.06 (0.86-1.31) | 0.82 (0.65-1.04) |
|  | *Surgery + adjuvant CHT* | *110* |  | *57 (51.8)* | *1.40 (1.05-1.86)* | *0.78 (0.57-1.05)* |  | *70 (63.6)* | *1.01 (0.78-1.30)* | *0.68 (0.52-0.89)* |
|  | *Surgery + adjuvant RT* | *52* |  | *29 (55.8)* | *1.52 (1.04-2.24)* | *1.48 (0.95-2.31)* |  | *38 (73.1)* | *1.18 (0.85-1.64)* | *1.36 (0.93-1.98)* |
| *CRC*, colorectal cancer; *HR*, hazard ratio; *CI*, confidence interval; *CHT*, chemotherapy; *RT*, radiotherapy; *SARIFA*, Stroma AReactive Invasion Front Areas.  ^a^Adjusted for age at diagnosis (years), sex (male, female), tumour location (colon, rectosigmoid, rectum), pTNM stage (II, III, IV, unknown), differentiation grade (well, moderate, poor/undifferentiated, unknown), and MMR status (proficient, deficient) | | | | | | | | | | |
