# Supplementary material for: Prognostic and Predictive Value of SARIFA-status Within Molecular Subgroups of Colorectal Cancer: Insights From the Netherlands Cohort Study
Source: Am J Surg Pathol. 2025 May 9;49(9):956–69. doi: 10.1097/PAS.0000000000002408 (PMC12352556; doi:10.1097/PAS.0000000000002408)
Supplement: Supplementary file 8 [file pas-49-956-s008.docx]

**Supplementary Table S7** – Association between adjuvant therapy and CRC-specific and overall survival of pT stage 3-4 colorectal cancer patients within the Netherlands Cohort Study (NLCS, 1986-2006), according to SARIFA status (SARIFA-positive and SARIFA-negative; *n* = 1,295).

|  | | **N** |  | **CRC-specific survival** | | |  | **Overall survival** | | |
| --- | --- | --- | --- | --- | --- | --- | --- | --- | --- | --- |
|  |  |  |  | **CRC deaths (%)** | **HR (95% CI)** | |  | **Deaths (%)** | **HR (95% CI)** | |
|  | |  |  |  | **Univariable** | **Multivariable-adjusted^a^** |  |  | **Univariable** | **Multivariable-adjusted^a^** |
| **Colorectal cancer** | |  |  |  |  |  |  |  |  |  |
|  | Surgery only | 1050 |  | 457 (43.5) | 1.00 (ref) | 1.00 (ref) |  | 712 (67.8) | 1.00 (ref) | 1.00 (ref) |
|  | Surgery + adjuvant therapy | 245 |  | 152 (62.0) | 1.45 (1.20-1.74) | 0.79 (0.65-0.97) |  | 187 (76.3) | 1.19 (1.01-1.39) | 0.77 (0.65-0.93) |
|  | *Surgery + adjuvant CHT* | *187* |  | *120 (64.2)* | *1.54 (1.26-1.89)* | *0.72 (0.57-0.89)* |  | *144 (77.0)* | *1.24 (1.04-1.48)* | *0.69 (0.57-0.84)* |
|  | *Surgery + adjuvant RT* | *58* |  | *32 (55.2)* | *1.18 (0.82-1.68)* | *1.32 (0.87-1.99)* |  | *43 (74.1)* | *1.03 (0.76-1.41)* | *1.26 (0.89-1.80)* |
|  |  |  |  |  |  |  |  |  |  |  |
| **SARIFA-positive** | |  |  |  |  |  |  |  |  |  |
|  | Surgery only | 358 |  | 215 (60.1) | 1.00 (ref) | 1.00 (ref) |  | 283 (79.1) | 1.00 (ref) | 1.00 (ref) |
|  | Surgery + adjuvant therapy | 108 |  | 78 (72.2) | 1.13 (0.87-1.47) | 0.60 (0.45-0.80) |  | 93 (86.1) | 1.07 (0.84-1.35) | 0.63 (0.49-0.82) |
|  | *Surgery + adjuvant CHT* | *94* |  | *72 (76.6)* | *1.24 (0.95-1.63)* | *0.61 (0.45-0.82)* |  | *83 (88.3)* | *1.14 (0.89-1.46)* | *0.62 (0.47-0.82)* |
|  | *Surgery + adjuvant RT* | *14* |  | *6 (42.9)* | *0.55 (0.25-1.25)* | *0.49 (0.19-1.28)* |  | *10 (71.4)* | *0.70 (0.37-1.31)* | *0.73 (0.35-1.51)* |
|  |  |  |  |  |  |  |  |  |  |  |
| **SARIFA-negative** | |  |  |  |  |  |  |  |  |  |
|  | Surgery only | 692 |  | 242 (35.0) | 1.00 (ref) | 1.00 (ref) |  | 429 (62.0) | 1.00 (ref) | 1.00 (ref) |
|  | Surgery + adjuvant therapy | 137 |  | 74 (54.0) | 1.56 (1.20-2.02) | 0.99 (0.74-1.34) |  | 94 (68.6) | 1.14 (0.91-1.43) | 0.87 (0.67-1.13) |
|  | *Surgery + adjuvant CHT* | *93* |  | *48 (51.6)* | *1.48 (1.08-2.02)* | *0.78 (0.55-1.10)* |  | *61 (65.6)* | *1.08 (0.83-1.41)* | *0.69 (0.51-0.94)* |
|  | *Surgery + adjuvant RT* | *44* |  | *26 (59.1)* | *1.73 (1.15-2.59)* | *1.95 (1.21-3.13)* |  | *33 (75.0)* | *1.28 (0.90-1.82)* | *1.59 (1.06-2.38)* |
| *CRC*, colorectal cancer; *HR*, hazard ratio; *CI*, confidence interval; *CHT*, chemotherapy; *RT*, radiotherapy; *SARIFA*, Stroma AReactive Invasion Front Areas.  ^a^Adjusted for age at diagnosis (years), sex (male, female), tumour location (colon, rectosigmoid, rectum), differentiation grade (well, moderate, poor/undifferentiated, unknown), and MMR status (proficient, deficient) | | | | | | | | | | |
