# Supplementary material for: Prognostic and Predictive Value of SARIFA-status Within Molecular Subgroups of Colorectal Cancer: Insights From the Netherlands Cohort Study
Source: Am J Surg Pathol. 2025 May 9;49(9):956–69. doi: 10.1097/PAS.0000000000002408 (PMC12352556; doi:10.1097/PAS.0000000000002408)
Supplement: Supplementary file 9 [file pas-49-956-s009.docx]

**Supplementary Table S8** – Association between adjuvant therapy and CRC-specific and overall survival of pT stage 3-4 colon cancer patients within the Netherlands Cohort Study (NLCS, 1986-2006), according to SARIFA status (SARIFA-positive and SARIFA-negative; *n* = 1,019).

|  | | **N** |  | **CRC-specific survival** | | |  | **Overall survival** | | |
| --- | --- | --- | --- | --- | --- | --- | --- | --- | --- | --- |
|  |  |  |  | **CRC deaths (%)** | **HR (95% CI)** | |  | **Deaths (%)** | **HR (95% CI)** | |
|  | |  |  |  | **Univariable** | **Multivariable-adjusted^a^** |  |  | **Univariable** | **Multivariable-adjusted^a^** |
| **Colon cancer** | |  |  |  |  |  |  |  |  |  |
|  | Surgery only | 862 |  | 386 (44.8) | 1.00 (ref) | 1.00 (ref) |  | 585 (67.9) | 1.00 (ref) | 1.00 (ref) |
|  | Surgery + adjuvant therapy | 157 |  | 96 (61.1) | 1.36 (1.09-1.70) | 0.65 (0.51-0.83) |  | 120 (76.4) | 1.16 (0.95-1.41) | 0.65 (0.52-0.81) |
|  | *Surgery + adjuvant CHT* | *150* |  | *93 (62.0)* | *1.40 (1.11-1.75)* | *0.64 (0.50-0.83)* |  | *115 (76.7)* | *1.18 (0.97-1.45)* | *0.64 (0.51-0.80)* |
|  | *Surgery + adjuvant RT* | *7* |  | *3 (42.9)* | *0.76 (0.24-2.37)* | *0.92 (0.29-2.88)* |  | *5 (71.4)* | *0.81 (0.34-1.96)* | *0.95 (0.39-2.31)* |
|  |  |  |  |  |  |  |  |  |  |  |
| **SARIFA-positive** | |  |  |  |  |  |  |  |  |  |
|  | Surgery only | 312 |  | 190 (60.9) | 1.00 (ref) | 1.00 (ref) |  | 246 (78.8) | 1.00 (ref) | 1.00 (ref) |
|  | Surgery + adjuvant therapy | 83 |  | 60 (72.3) | 1.08 (0.81-1.44) | 0.56 (0.41-0.77) |  | 71 (85.5) | 1.02 (0.78-1.32) | 0.57 (0.43-0.76) |
|  | *Surgery + adjuvant CHT* | *81* |  | *59 (72.8)* | *1.10 (0.82-1.48)* | *0.56 (0.41-0.78)* |  | *70 (86.4)* | *1.05 (0.80-1.37)* | *0.57 (0.43-0.77)* |
|  | *Surgery + adjuvant RT* | *2* |  | *1 (50.0)* | *0.45 (0.06-3.22)* | *0.54 (0.07-3.95)* |  | *1 (50.0)* | *0.33 (0.05-2.36)* | *0.39 (0.05-2.80)* |
|  |  |  |  |  |  |  |  |  |  |  |
| **SARIFA-negative** | |  |  |  |  |  |  |  |  |  |
|  | Surgery only | 550 |  | 196 (35.6) | 1.00 (ref) | 1.00 (ref) |  | 339 (61.6) | 1.00 (ref) | 1.00 (ref) |
|  | Surgery + adjuvant therapy | 74 |  | 36 (48.6) | 1.33 (0.93-1.89) | 0.68 (0.46-1.02) |  | 49 (66.2) | 1.05 (0.78-1.42) | 0.66 (0.47-0.93) |
|  | *Surgery + adjuvant CHT* | *69* |  | *34 (49.3)* | *1.35 (0.94-1.94)* | *0.66 (0.44-1.00)* |  | *45 (65.2)* | *1.04 (0.76-1.42)* | *0.63 (0.44-0.89)* |
|  | *Surgery + adjuvant RT* | *5* |  | *2 (40.0)* | *1.05 (0.26-4.22)* | *1.17 (0.29-4.74)* |  | *4 (80.0)* | *1.19 (0.44-3.19)* | *1.30 (0.48-3.52)* |
| *CRC*, colorectal cancer; *HR*, hazard ratio; *CI*, confidence interval; *CHT*, chemotherapy; *RT*, radiotherapy; *SARIFA*, Stroma AReactive Invasion Front Areas.  ^a^Adjusted for age at diagnosis (years), sex (male, female), differentiation grade (well, moderate, poor/undifferentiated, unknown), pTNM stage (I/II/III/IV) and MMR status (proficient, deficient) | | | | | | | | | | |
