# Supplementary material for: Prognostic and Predictive Value of SARIFA-status Within Molecular Subgroups of Colorectal Cancer: Insights From the Netherlands Cohort Study
Source: Am J Surg Pathol. 2025 May 9;49(9):956–69. doi: 10.1097/PAS.0000000000002408 (PMC12352556; doi:10.1097/PAS.0000000000002408)
Supplement: Supplementary file 10 [file pas-49-956-s010.docx]

**Supplementary Table S9** – Association between adjuvant therapy and CRC-specific and overall survival of pTNM stage II colorectal cancer patients within the Netherlands Cohort Study (NLCS, 1986-2006), according to SARIFA status (SARIFA-positive and SARIFA-negative; *n* = 655).

|  | | **N** |  | **CRC-specific survival** | | |  | **Overall survival** | | |
| --- | --- | --- | --- | --- | --- | --- | --- | --- | --- | --- |
|  |  |  |  | **CRC deaths (%)** | **HR (95% CI)** | |  | **Deaths (%)** | **HR (95% CI)** | |
|  | |  |  |  | **Univariable** | **Multivariable-adjusted^a^** |  |  | **Univariable** | **Multivariable-adjusted^a^** |
| **Colorectal cancer** | |  |  |  |  |  |  |  |  |  |
|  | Surgery only | 610 |  | 158 (25.9) | 1.00 (ref) | 1.00 (ref) |  | 334 (54.8) | 1.00 (ref) | 1.00 (ref) |
|  | Surgery + adjuvant therapy | 45 |  | 17 (37.8) | 1.46 (0.88-2.40) | 1.48 (0.85-2.59) |  | 27 (60.0) | 1.09 (0.74-1.62) | 1.37 (0.89-2.10) |
|  | *Surgery + adjuvant CHT* | *15* |  | *3 (20.0)* | *0.68 (0.22-2.13)* | *0.76 (0.24-2.42)* |  | *6 (40.0)* | *0.61 (0.27-1.37)* | *0.76 (0.33-1.71)* |
|  | *Surgery + adjuvant RT* | *30* |  | *14 (46.7)* | *1.93 (1.12-3.34)* | *1.99 (1.04-3.80)* |  | *21 (70.0)* | *1.41 (0.91-2.19)* | *1.91 (1.15-3.19)* |
|  |  |  |  |  |  |  |  |  |  |  |
| **SARIFA-positive** | |  |  |  |  |  |  |  |  |  |
|  | Surgery only | 141 |  | 55 (39.0) | 1.00 (ref) | 1.00 (ref) |  | 89 (63.1) | 1.00 (ref) | 1.00 (ref) |
|  | Surgery + adjuvant therapy | 9 |  | 3 (33.3) | 0.79 (0.25-2.52) | 0.88 (0.26-2.93) |  | 6 (66.7) | 0.96 (0.42-2.19) | 0.95 (0.40-2.25) |
|  | *Surgery + adjuvant CHT* | *4* |  | *2 (50.0)* | *1.27 (0.31-5.20)* | *1.39 (0.33-5.94)* |  | *3 (75.0)* | *1.14 (0.36-3.60)* | *1.07 (0.33-3.45)* |
|  | *Surgery + adjuvant RT* | *5* |  | *1 (20.0)* | *0.45 (0.06-3.23)* | *0.48 (0.06-3.77)* |  | *3 (60.0)* | *0.82 (0.26-2.60)* | *0.84 (0.24-2.91)* |
|  |  |  |  |  |  |  |  |  |  |  |
| **SARIFA-negative** | |  |  |  |  |  |  |  |  |  |
|  | Surgery only | 469 |  | 103 (22.0) | 1.00 (ref) | 1.00 (ref) |  | 245 (52.2) | 1.00 (ref) | 1.00 (ref) |
|  | Surgery + adjuvant therapy | 36 |  | 14 (38.9) | 1.81 (1.03-3.16) | 1.74 (0.91-3.32) |  | 21 (58.3) | 1.14 (0.73-1.78) | 1.48 (0.90-2.43) |
|  | *Surgery + adjuvant CHT* | *11* |  | *1 (9.1)* | *0.35 (0.05-2.50)* | *0.43 (0.06-3.16)* |  | *3 (27.3)* | *0.42 (0.13-1.30)* | *0.58 (0.18-1.85)* |
|  | *Surgery + adjuvant RT* | *25* |  | *13 (52.0)* | *2.67 (1.50-4.75)* | *2.45 (1.21-4.98)* |  | *18 (72.0)* | *1.61 (0.99-2.59)* | *2.14 (1.22-3.79)* |
| *CRC*, colorectal cancer; *HR*, hazard ratio; *CI*, confidence interval; *CHT*, chemotherapy; *RT*, radiotherapy; *SARIFA*, Stroma AReactive Invasion Front Areas.  ^a^Adjusted for age at diagnosis (years), sex (male, female), tumour location (colon, rectosigmoid, rectum), differentiation grade (well, moderate, poor/undifferentiated, unknown), and MMR status (proficient, deficient) | | | | | | | | | | |
