# Supplementary material for: Prognostic and Predictive Value of SARIFA-status Within Molecular Subgroups of Colorectal Cancer: Insights From the Netherlands Cohort Study
Source: Am J Surg Pathol. 2025 May 9;49(9):956–69. doi: 10.1097/PAS.0000000000002408 (PMC12352556; doi:10.1097/PAS.0000000000002408)
Supplement: Supplementary file 11 [file pas-49-956-s011.docx]

**Supplementary Table S10** – Association between adjuvant therapy and CRC-specific and overall survival of pTNM stage II colon cancer patients within the Netherlands Cohort Study (NLCS, 1986-2006), according to SARIFA status (SARIFA-positive and SARIFA-negative; *n* = 518).

| **S** | | **N** |  | **CRC-specific survival** | | |  | **Overall survival** | | |
| --- | --- | --- | --- | --- | --- | --- | --- | --- | --- | --- |
|  |  |  |  | **CRC deaths (%)** | **HR (95% CI)** | |  | **Deaths (%)** | **HR (95% CI)** | |
|  | |  |  |  | **Univariable** | **Multivariable-adjusted^a^** |  |  | **Univariable** | **Multivariable-adjusted^a^** |
| **Colon cancer** | |  |  |  |  |  |  |  |  |  |
|  | Surgery only | 501 |  | 134 (26.7) | 1.00 (ref) | 1.00 (ref) |  | 272 (54.3) | 1.00 (ref) | 1.00 (ref) |
|  | Surgery + adjuvant therapy | 17 |  | 6 (35.3) | 1.28 (0.56-2.89) | 1.30 (0.57-2.99) |  | 11 (64.7) | 1.14 (0.63-2.09) | 1.30 (0.70-2.39) |
|  | *Surgery + adjuvant CHT* | *12* |  | *3 (25.0)* | *0.86 (0.27-2.70)* | *0.93 (0.29-2.98)* |  | *6 (50.0)* | *0.82 (0.36-1.83)* | *0.95 (0.42-2.14)* |
|  | *Surgery + adjuvant RT* | *5* |  | *3 (60.0)* | *2.49 (0.79-7.84)* | *2.12 (0.67-6.72)* |  | *5 (100.0)* | *2.21 (0.91-5.35)* | *2.30 (0.94-5.61)* |
|  |  |  |  |  |  |  |  |  |  |  |
| **SARIFA-positive** | |  |  |  |  |  |  |  |  |  |
|  | Surgery only | 125 |  | 49 (39.2) | 1.00 (ref) | 1.00 (ref) |  | 77 (61.6) | 1.00 (ref) | 1.00 (ref) |
|  | Surgery + adjuvant therapy | 5 |  | 3 (60.0) | 1.42 (0.44-4.56) | 1.36 (0.41-4.48) |  | 4 (80.0) | 1.21 (0.44-3.30) | 1.09 (0.39-3.04) |
|  | *Surgery + adjuvant CHT* | *4* |  | *2 (50.0)* | *1.26 (0.31-5.17)* | *1.44 (0.34-6.19)* |  | *3 (75.0)* | *1.16 (0.36-3.67)* | *1.06 (0.33-3.45)* |
|  | *Surgery + adjuvant RT* | *1* |  | *1 (100.0)* | *1.92 (0.26-13.92)* | *1.23 (0.16-9.20)* |  | *1 (100.0)* | *1.40 (0.19-10.11)* | *1.17 (0.16-8.72)* |
|  |  |  |  |  |  |  |  |  |  |  |
| **SARIFA-negative** | |  |  |  |  |  |  |  |  |  |
|  | Surgery only | 376 |  | 85 (22.6) | 1.00 (ref) | 1.00 (ref) |  | 195 (51.9) | 1.00 (ref) | 1.00 (ref) |
|  | Surgery + adjuvant therapy | 12 |  | 3 (25.0) | 1.07 (0.34-3.39) | 1.19 (0.37-3.82) |  | 7 (58.3) | 1.06 (0.50-2.26) | 1.35 (0.63-2.90) |
|  | *Surgery + adjuvant CHT* | *8* |  | *1 (12.5)* | *0.49 (0.07-3.52)* | *0.58 (0.08-4.22)* |  | *3 (37.5)* | *0.61 (0.19-1.90)* | *0.82 (0.26-2.61)* |
|  | *Surgery + adjuvant RT* | *4* |  | *2 (50.0)* | *2.65 (0.65-10.76)* | *2.43 (0.59-9.97)* |  | *4 (100.0)* | *2.40 (0.89-6.47)* | *2.52 (0.93-6.84)* |
| *CRC*, colorectal cancer; *HR*, hazard ratio; *CI*, confidence interval; *CHT*, chemotherapy; *RT*, radiotherapy; *SARIFA*, Stroma AReactive Invasion Front Areas.  ^a^Adjusted for age at diagnosis (years), sex (male, female), differentiation grade (well, moderate, poor/undifferentiated, unknown), and MMR status (proficient, deficient) | | | | | | | | | | |
